# Supplementary material for: Spatial coalescent connectivity through multi-generation dispersal modelling predicts gene flow across marine phyla
Source: Nat Commun. 2022 Oct 4;13:5861. doi: 10.1038/s41467-022-33499-z (PMC9532449; doi:10.1038/s41467-022-33499-z)
Supplement: Supplementary file 1 — Supplementary Information [file 41467_2022_33499_MOESM1_ESM.pdf]

## Supplementary Information:

# Spatial coalescent connectivity through multi-generation dispersal modelling predicts gene flow across marine phyla

Térence Legrand[1]*; Anne Chenuil[2]; Enrico Ser-Giacomi[3]; Sophie Arnaud-Haond[4]; Nicolas Bierne[5]; Vincent Rossi[6]*

## Affiliations :

[1]Aix Marseille University, Universite de Toulon, CNRS, IRD, Mediterranean Institute of Oceanography (UMR 7294), Marseille, France. (legrandterence@gmail.com).

[2] Institut Méditerranéen de Biodiversité et d'Ecologie Marine et Continentale, CNRS (UMR 7263), Station Marine d'Endoume, Marseille, France. (anne.chenuil@imbe.fr).

[3]Department of Earth, Atmospheric and Planetary Sciences, Massachusetts Institute of Technology, 54-1514 MIT, Cambridge, Massachusetts, USA. (enrico.sergiacomi@gmail.com).

[4] MARBEC (Marine Biodiversity, Exploitation and Conservation, UMR 9190) Univ. Montpellier, IFREMER, IRD, CNRS, Sète, France. (sophie.arnaud-haond@umontpellier.fr).

[5]ISEM, Univ Montpellier, CNRS, IRD, Montpellier, France. (nicolas.bierne@umontpellier.fr).

[6]Aix Marseille University, Universite de Toulon, CNRS, IRD, Mediterranean Institute of Oceanography (UMR 7294), Marseille, France (vincent.rossi@mio.osupytheas.fr).

*Corresponding authors

# Supplementary Methods 1: Selected references, model parameterization and literature review

*Supplementary Table 1:* ***Summarized description of the 58 population genetic studies included in the meta-analysis****. Note that some results reported by a given study are analysed separately: (i) Weber et al., 2015 used SNPs marker (*[1]*) and mtDNA marker (*[2]*); (ii) Carrera et al., 2020 considered all the loci together (*[3]*) and then only the Mediterranean outliers loci (*[4]*); (iii) Marzouk et al., 2017 analyzed SNPs marker (*[5]*) and mtDNA marker (*[6]*). Dbtw is the mean straight-line geographical distance (in km) between sampled localities. Source data are provided as a Source Data file.*

| Species characteristics | | | | | Study characteristics | | | |
|---|---|---|---|---|---|---|---|---|
| **Taxa** | **Species** | **Habitat** | **PLD** | **Season** | **Study** | **Marker** | **Nbr of populations** | **$D_{btw}$** |
| Algea | Cystoseira amentacea | shallow coastal | 1 | all | Susini et al., 2007 | RAPD | 4 | 223 |
| Anthozoa | Astroides calycularis | shallow coastal | 1 | summer | Casado-Amezua et al., 2012 | microsat | 16 | 617 |
| Anthozoa | Corallium rubrum | neritic shelf | 10 | summer | Aurelle et al., 2011 | microsat | 24 | 729 |
| Anthozoa | Corallium rubrum | neritic shelf | 10 | summer | Costantini et al., 2013 | microsat | 5 | 716 |
| Anthozoa | Eunicella cavolinii | shallow coastal | 1 | all | Masmoudi et al., 2016 | microsat | 18 | 715 |
| Anthozoa | Leptopsammia pruvoti | shallow coastal | 10 | spring | Boscari et al., 2019 | SNP/RAD_GBS_transc | 10 | 793 |
| Ascidiacea | Botryllus schlosseri | shallow coastal | 1 | all | Reem et al., 2017 | microsat | 11 | 1020 |
| Ascidiacea | Halocynthia papillosa | neritic shelf | 20 | summer | Villamor et al., 2014 | mtDNA | 4 | 1018 |
| Ascidiacea | Microcosmus squamiger | shallow coastal | 1 | summer | Ordóñez et al., 2013 | microsat | 6 | 970 |
| Ascidiacea | Pycnoclavella communis | shallow coastal | 1 | all | Pérez-Portela et al., 2007 | microsat | 4 | 983 |
| Crustacea | Carcinus aestuarii | shallow coastal | 30 | fall | Schiavina et al., 2014 | microsat | 8 | 965 |
| Crustacea | Melicertus kerathurus | shallow coastal | 20 | all | Arculeo et al., 2010 | microsat | 9 | 996 |
| Crustacea | Melicertus kerathurus | shallow coastal | 20 | all | Zitari-Chatti et al., 2007 | allozymes | 9 | 911 |
| Crustacea | Pachygrapsus marmoratus | shallow coastal | 30 | all | Fratini et al., 2013 | microsat | 8 | 896 |
| Crustacea | Palinurus elephas | shallow coastal | 45 | spring | Palero et al., 2011 | microsat | 5 | 945 |
| Demospongiae | Spongia officinalis | shallow coastal | 1 | summer | Dailianis et al., 2011 | microsat | 9 | 1059 |
| Echinodermata | Astropecten aranciacus | shallow coastal | 45 | all | Zulliger et al., 2009 | microsat | 7 | 1076 |
| Echinodermata | Holothuria mammata | shallow coastal | 20 | spring | Borrero-Pérez et al., 2011 | microsat | 4 | 1087 |
| Echinodermata | Ophioderma longicauda | shallow coastal | 10 | spring | Weber et al., 2015[1] | mtDNA | 13 | 1047 |

| Species characteristics | | | | | Study characteristics | | | |
|---|---|---|---|---|---|---|---|---|
| **Taxa** | **Species** | **Habitat** | **PLD** | **Season** | **Study** | **Marker** | **Nbr of populations** | **$D_{btw}$** |
| Echinodermata | Ophioderma longicauda | shallow coastal | 10 | spring | Weber et al., 2015[2] | nuclearDNAseq | 11 | 1127 |
| Echinodermata | Paracentrotus lividus | shallow coastal | 30 | spring | Penant et al., 2013 | mtDNA | 12 | 1134 |
| Echinodermata | Paracentrotus lividus | shallow coastal | 30 | spring | Paterno et al., 2017 | SNP/RAD_GBS_transc | 10 | 1094 |
| Echinodermata | Paracentrotus lividus | shallow coastal | 30 | spring | Carreras et al., 2020[3] | SNP/RAD_GBS_transc | 8 | 1183 |
| Echinodermata | Paracentrotus lividus | shallow coastal | 30 | spring | Carreras et al., 2020[4] | SNP/RAD_GBS_transc | 8 | 1183 |
| Fish | Apogon imberbis | shallow coastal | 20 | summer | Muths et al., 2015 | microsat | 4 | 1178 |
| Fish | Coris julis | shallow coastal | 30 | summer | Fruciano et al., 2011 | mtDNA | 10 | 1144 |
| Fish | Diplodus sargus | shallow coastal | 20 | spring | González-Wangüemert et al., 2010 | microsat | 5 | 1141 |
| Fish | Diplodus vulgaris | shallow coastal | 45 | winter | Kaouèche et al., 2013 | allozymes | 6 | 1103 |
| Fish | Epinephelus marginatus | shallow coastal | 30 | summer | Schunter et al., 2011 | microsat | 9 | 1149 |
| Fish | Lithognathus mormyrus | shallow coastal | 30 | all | Hammami et al., 2007 | allozymes | 4 | 1144 |
| Fish | Merluccius merluccius | neritic shelf | 45 | all | Milano et al., 2014 | SNP/RAD_GBS_transc | 14 | 1147 |
| Fish | Mugil cephalus | shallow coastal | 45 | all | Durand et al., 2013 | microsat | 12 | 1279 |
| Fish | Mullus barbartus | shallow coastal | 30 | spring | Maggio et al., 2009 | microsat | 14 | 882 |
| Fish | Mullus surmuletus | shallow coastal | 30 | spring | Galarza et al., 2009 | microsat | 6 | 919 |
| Fish | Mullus surmuletus | shallow coastal | 30 | spring | Dalongeville et al., 2018 | SNP/RAD_GBS_transc | 47 | 1559 |
| Fish | Oblada melanura | shallow coastal | 20 | spring | Gkafas et al., 2013 | microsat | 8 | 1555 |
| Fish | Oblada melanura | shallow coastal | 20 | spring | Calò et al., 2016 | microsat | 9 | 1561 |
| Fish | Pagellus erythrinus | shallow coastal | 45 | spring | Fassatoui et al., 2009 | allozymes | 6 | 1561 |
| Fish | Serranus cabrilla | shallow coastal | 30 | spring | Schunter et al., 2011 | microsat | 13 | 1592 |
| Fish | Solea solea | shallow coastal | 30 | winter | Bahri-Sfar et al., 2011 | allozymes | 10 | 1603 |
| Fish | Solea solea | shallow coastal | 30 | winter | Garoia et al., 2007 | microsat | 4 | 1607 |
| Fish | Sparus aurata | shallow coastal | 45 | fall | Franchini et al., 2012 | microsat | 12 | 1569 |
| Fish | Symphodus tinca | shallow coastal | 10 | spring | Carreras et al., 2017 | SNP/RAD_GBS_transc | 6 | 1569 |
| Mollusca | Cerastoderma edule | shallow coastal | 10 | all | Sromek et al., 2019 | SNP/RAD_GBS_transc | 7 | 1583 |
| Mollusca | Chiton olivaceus | shallow coastal | 10 | spring | Villamor et al., 2014 | mtDNA | 4 | 1583 |

| Species characteristics | | | | | Study characteristics | | | |
|---|---|---|---|---|---|---|---|---|
| **Taxa** | **Species** | **Habitat** | **PLD** | **Season** | **Study** | **Marker** | **Nbr of populations** | **$D_{btw}$** |
| Mollusca | Hexaplex trunculus | shallow coastal | 1 | all | Villamor et al., 2014 | mtDNA | 4 | 1583 |
| Mollusca | Hexaplex trunculus | shallow coastal | 1 | all | Marzouk et al., 2017[5] | nuclearDNAseq | 15 | 1627 |
| Mollusca | Hexaplex trunculus | shallow coastal | 1 | all | Marzouk et al., 2017[6] | mtDNA | 15 | 1627 |
| Mollusca | Mytilus galloprovincialis | shallow coastal | 20 | spring | Diz and Presa, 2008 | microsat | 8 | 1609 |
| Mollusca | Ostrea edulis | shallow coastal | 10 | summer | Launey et al., 2002 | microsat | 5 | 1617 |
| Mollusca | Patella caerulea | shallow coastal | 20 | fall | Villamor et al., 2014 | mtDNA | 6 | 1612 |
| Mollusca | Patella rustica | shallow coastal | 20 | fall | Sá-Pinto et al., 2012 | allozymes | 6 | 1623 |
| Mollusca | Patella ulyssiponensis | shallow coastal | 20 | summer | Sá-Pinto et al., 2012 | allozymes | 5 | 1624 |
| Mollusca | Phorcus turbinatus | shallow coastal | 10 | all | Villamor et al., 2014 | mtDNA | 4 | 1620 |
| Mollusca | Ruditapes decussatus | shallow coastal | 10 | summer | Gharbi et al., 2011 | allozymes | 11 | 1589 |
| Mollusca | Spondylus spinosus | shallow coastal | 20 | all | Shabtay et al., 2014 | mtDNA | 5 | 1587 |
| Phanerogam | Cymodocea nodosa | shallow coastal | 30 | all | Alberto et al., 2008 | microsat | 13 | 1601 |
| Phanerogam | Posidonia oceanica | shallow coastal | 30 | all | Arnaud-Haond et al., 2007 | microsat | 29 | 1588 |

*Supplementary Table 2: Literature reference used to configure species characteristics (i.e. habitat, PLD, spawning season). We also use FishBase (Froese and Pauly 2000, https://www.fishbase.se/search.php) and Doris (Willis et al., 2016, https://doris.ffessm.fr/) webpages for global information about the species of interest.*

| Species | References |
|---|---|
| *Apogon imberbis* | (Macpherson and Raventos, 2006; Raventos, 2007) |
| *Astroides calycularis* | (Casado-Amezúa et al., 2012; Goffredo et al., 2010) |
| *Astropecten aranciacus* | (Baeta et al., 2016; Zulliger et al., 2009) |
| *Balanophyllia europaea* | (Goffredo et al., 2004) |
| *Botryllus schlosseri* | (Reem et al., 2017) |
| *Perforatus perforatus* | (Villamor et al., 2014) |
| *Carcinus aestuarii* | (Carlton and Cohen, 2003; Schiavina et al., 2014) |
| *Cerastoderma edule* | (Boyden and Russell, 1972) |
| *Chiton olivaceus* | (Villamor et al., 2014; Wanninger and Haszprunar, 2002) |
| *Chondrosia reniformis* | (Lazoski et al., 2001; Villamor et al., 2014) |
| *Cladocora caespitosa* | (Casado-Amezúa et al., 2012; Kersting et al., 2013; Kružić et al., 2008) |
| *Corallium rubrum* | (Coelho and Lasker, 2016; Costantini et al., 2013; Teixidó et al., 2011) |
| *Coris julis* | (Fruciano et al., 2011; Macpherson and Raventos, 2006) |
| *Crassostrea gigas* | (Ernande et al., 2003) |
| *Cymodocea nodosa* | (Alberto et al., 2008; Orth et al., 2006) |
| *Cystoseira amentacea* | (Susini et al., 2007; Thibaut et al., 2016) |
| *Diplodus puntazzo* | (Di Franco and Guidetti, 2011) |
| *Diplodus sargus* | (Di Franco et al., 2013) |
| *Diplodus vulgaris* | (Di Franco et al., 2013; Macpherson and Raventos, 2006) |
| *Eunicella cavolinii* | (Cánovas-Molina et al., 2018; Masmoudi et al., 2016) |
| *Fistularia commersonii* | (Bernardi et al., 2016) |
| *Epinephelus marginatus* | (Macpherson and Raventos, 2006) |
| *Halocynthia papillosa* | (Villamor et al., 2014) |
| *Hexaplex trunculus* | (Vasconcelos et al., 2004) |
| *Holothuria mammata* | (Borrero-Pérez et al., 2011; Santos et al., n.d.) |
| *Leptopsammia pruvoti* | (Boscari et al., 2019; Goffredo et al., 2006) |
| *Lithognathus mormyrus* | Fishbase & Doris |
| *Melicertus kerathurus* | (Arculeo et al., 2010; Roberts et al., 2012; Zitari-Chatti et al., 2007) |
| *Merluccius merluccius* | (Hidalgo et al., 2019; Morales-Nin and Moranta, 2004) |
| *Microcosmus squamiger* | (Rius et al., 2010, 2009) |
| *Mugil cephalus* | (Kuo et al., 1973) |
| *Mullus barbartus* | (Félix-Hackradt et al., 2013; Maggio et al., 2009) |
| *Mullus surmuletus* | (Félix-Hackradt et al., 2013; Macpherson and Raventos, 2006) |
| *Mytilus galloprovincialis* | (Caceres-Martinez et al., 1993) |
| *Oblada melanura* | (Macpherson and Raventos, 2006) |
| *Phorcus turbinatus* | (Villamor et al., 2014) |
| *Ophioderma longicauda* | (Weber et al., 2014, 2015) |
| *Ostrea edulis* | (Bierne et al., 1998) |
| *Pachygrapsus marmoratus* | (Cuesta and Rodríguez, 2000) |
| *Pagellus erythrinus* | (Macpherson and Raventos, 2006) |
| *Palinurus elephas* | (Hunter, 1999) |
| *Paracentrotus lividus* | (Pedrotti, 1993) |

| Species | References |
|---|---|
| *Patella caerulea* | (Villamor et al., 2014) |
| *Patella rustica* | (Sá-Pinto et al., 2012) |
| *Patella ulyssiponensis* | (Sá-Pinto et al., 2012) |
| *Posidonia oceanica* | (Melià et al., 2016; Serra et al., 2010) |
| *Pycnoclavella communis* | (Pérez-Portela et al., 2007) |
| *Ruditapes decussatus* | (Gharbi et al., 2011) |
| *Scopalina lophyropoda* | (Garoia et al., 2004) |
| *Serranus cabrilla* | (Macpherson and Raventos, 2006) |
| *Solea solea* | (Bahri-Sfar et al., 2011; Vaz et al., 2019) |
| *Sparus aurata* | (Franchini et al., 2012) |
| *Spondylus spinosus* | (Soria et al., 2010) |
| *Spongia officinalis* | (Baldacconi et al., 2007; Gaino et al., 2007) |
| *Symphodus tinca* | (Macpherson and Raventos, 2006; Pallaoro and Jardas, 2003) |

# Supplementary Notes 1: PRISMA 2020 flow diagram for updated systematic reviews

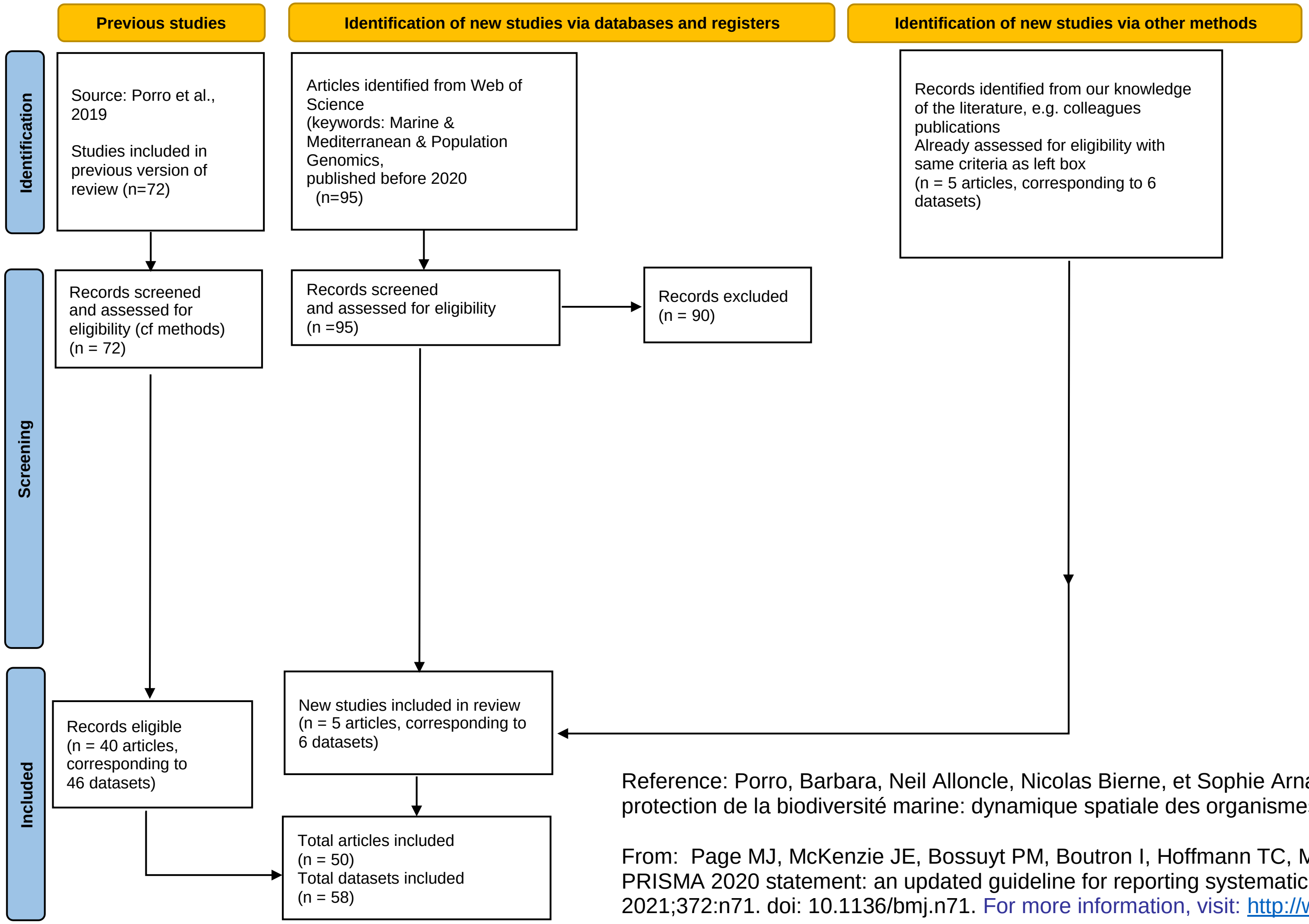


Reference: Porro, Barbara, Neil Alloncle, Nicolas Bierne, et Sophie Arnaud-Haond. Connectivité et protection de la biodiversité marine: dynamique spatiale des organismes marins. Quae, 2019

From: Page MJ, McKenzie JE, Bossuyt PM, Boutron I, Hoffmann TC, Mulrow CD, et al. The PRISMA 2020 statement: an updated guideline for reporting systematic reviews. BMJ 2021;372:n71. doi: 10.1136/bmj.n71. For more information, visit: http://www.prisma-statement.org/

# Supplementary Methods 2: Shallow coastal and neritic shelf habitat description

## Species-specific habitat

Based on the compiled literature of Supplementary Table 2, we attribute to each species pertaining to our meta-analysis (Supplementary Table 1) one habitat chosen between two broad types: shallow coastal habitat or neritic shelf habitat. If a species could fall into both habitat types, we select only one by (i) for fishes, retaining the habitat that contains the depth where the post-settlers/juveniles inhabit; (ii) for other organisms, retaining the habitat that comprises the most frequent sampling depths.

## Network habitat filter

### i. Bathymetric filter

Gridded bathymetry data provided by ETOPO1 1 Arc-Minute Global Relief Model (doi:10.7289/V5C8276M) are co-located with on our network grid to select all the topographic values encompassed in each node. Nodes whose shallowest depth is between 0 m and 50 m are kept in the shallow coastal bathymetric filter and those whose shallowest depth is between 50 m and 200 m are filtered in our neritic shelf bathymetric filter; the remaining deeper nodes are excluded.

### ii. Substrate filter

We use EMODnet Seabed Habitats data[1] to define our substrate filter combining both the EUNIS classification and the MSFD Benthic Broad Habitat typology. We distinguish *infralittoral* and *circalittoral* substrate categories, while considering all substrates together including rocks, fine muds, coarse sediments, etc. (Supplementary Table 3). By co-locating substrate data on our network grid, nodes which encompass *infralittoral* substrate are filtered in our shallow coastal substrate filter and nodes which encompass *circalittoral* substrate are filtered in our neritic shelf substrate filter.

*Supplementary Table 3:* ***Substrate typology used to define the two composite substrates****, called shallow coastal and neritic shelf substrates, following EUNIS and MFSD classifications referenced in EMODnet Seabed Habitats data.*

| Classifications | Shallow coastal substrate | Neritic shelf substrate |
|---|---|---|
| EUNIS | -'A3: Infralittoral rock and other hard substrata'<br>-'A4.26 or A4.32: Mediterranean coralligenous communities moderately exposed to hydrodynamic action or Mediterranean coralligenous communities sheltered from hydrodynamic action' | -'A4.26 or A4.32: Mediterranean coralligenous communities moderately exposed to hydrodynamic action or Mediterranean coralligenous communities sheltered from hydrodynamic action'<br>-'A4.27: Faunal communities on deep moderate energy circalittoral rock' |

[1] *Information contained here has been derived from data that is made available under the European Marine Observation Data Network (EMODnet) Seabed Habitats initiative (http://www.emodnet-seabedhabitats.eu/), financed by the European Union under Regulation (EU) No 508/2014 of the European Parliament and of the Council of 15 May 2014 on the European Maritime and Fisheries Fund.*

| Classifications | Shallow coastal substrate | Neritic shelf substrate |
|---|---|---|
| | -'A5.13: Infralittoral coarse sediment'<br>-'A5.23 or A5.33 or A5.34: Infralittoral fine sands or Infralittoral sandy mud or Infralittoral fine mud'<br>-'A5.23: Infralittoral fine sands'<br>-'A5.33: Infralittoral sandy mud'<br>-'A5.34: Infralittoral fine mud'<br>-'A5.5353: Facies of dead "mattes" of [Posidonia oceanica]'<br>-'A5.535: [Posidonia] beds' | -'A4: Circalittoral rock and other hard substrata<br>-'A5.14: Circalittoral coarse sediment'<br>-'A5.25: Circalittoral fine sand'<br>-'A5.26: Circalittoral muddy sand'<br>-'A5.35: Circalittoral sandy mud'<br>-'A5.36: Circalittoral fine mud' |
| MFSD | -'Infralittoral coarse sediment'<br>-'Infralittoral mixed sediment'<br>-'Infralittoral mud'<br>-'Infralittoral rock and biogenic reef'<br>-'Infralittoral sand' | -'Circalittoral coarse sediment'<br>-'Circalittoral mixed sediment'<br>-'Circalittoral mud'<br>-'Circalittoral mud or Offshore circalittoral mud'<br>-'Circalittoral rock and biogenic reef'<br>-'Circalittoral sand'<br>-'Offshore circalittoral coarse sediment'<br>-'Offshore circalittoral mixed sediment'<br>-'Offshore circalittoral mud'<br>-'Offshore circalittoral rock and biogenic reef'<br>-'Offshore circalittoral sand' |

1.

### iii. Habitat filter

The final habitat filter consists in the superposition of both bathymetric and substrate filters: coloured nodes characterize the shallow coastal habitat (Supplementary Figure 1 and Figure 1b main manuscript) and the neritic shelf habitat (Supplementary Figure 2, Figure 1c main manuscript). All nodes selected in the bathymetric filter are coloured in transparent light grey, those belonging to the substrate filter are displayed in red, while pink nodes represent the superposition of both filters.

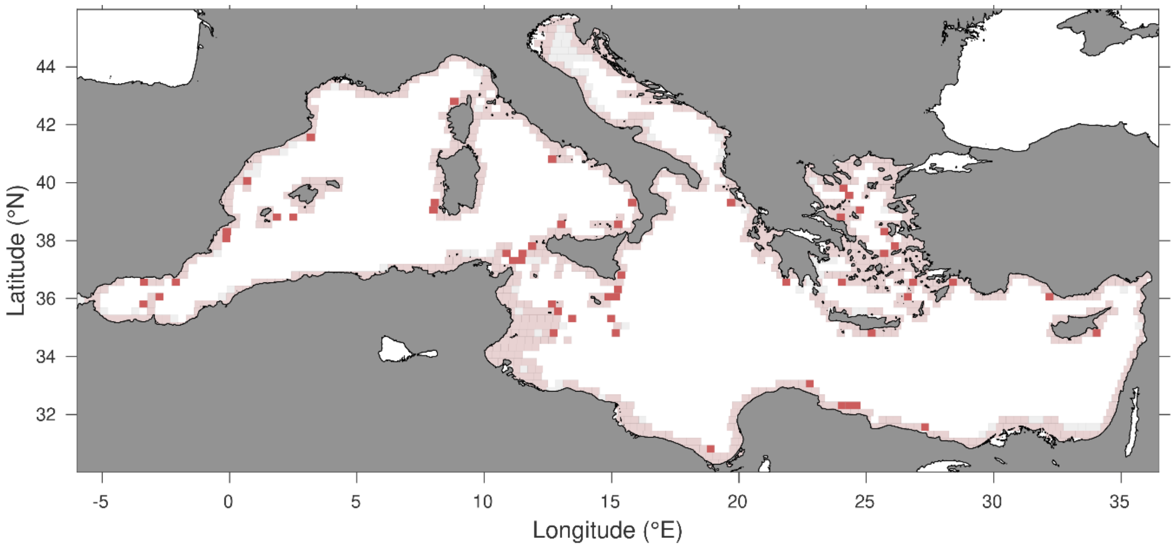


*Supplementary Figure 1:* ***Shallow coastal habitat*** *represented by a bathymetric filter (light grey nodes) and a substrate filter (red nodes). Pink nodes characterize the superposition of both bathymetric and substrate filters.*

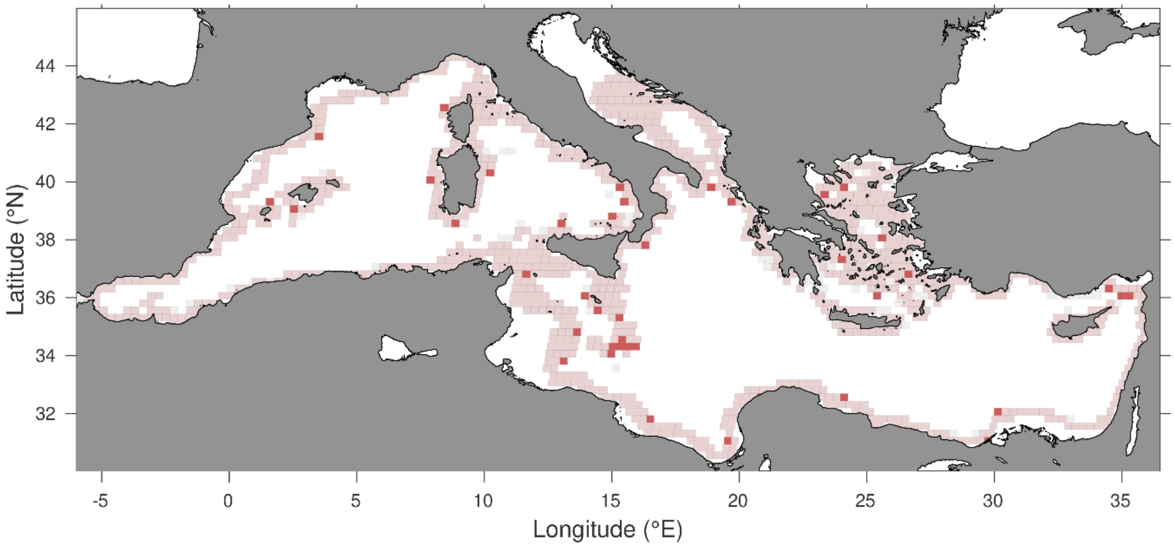


*Supplementary Figure 2:* ***Neritic shelf habitat*** *represented by a bathymetric filter (light grey nodes) and a substrate filter (red nodes). Pink nodes characterize the superposition of both bathymetric and substrate filters.*

# Supplementary Methods 3: Bio-physical modelling

## Hydrodynamical model

We use the hydrodynamic model Mediterranean Forecasting System (MFS) based on NEMO-OPA (Nucleus for European Modelling of the Ocean-PArellelis, version 3.2; Madec, 2008). This data-assimilative operational model has been implemented in the Mediterranean at 1/16° degree horizontal regular resolution and 72 unevenly spaced verticals levels (Oddo et al., 2009). We use the physics reanalysis products for years spanning 2000-2010 downloaded from Marine Copernicus website (https://marine.copernicus.eu/).

## Lagrangian modelling

Following the procedure described in Ser-Giacomi et al., (2015), we discretise the Mediterranean basin on a network of 8196 nodes of 1/4° degree horizontal resolution. About 100 numerical particles are evenly initialised in each node. Horizontal trajectories are simulated by integrating the velocity field, bilinearly interpolated using a *Runge-Kutta* 4 algorithm with a time step of 0.3 h, fulfilling the Courant-Friedrichs-Lewy condition (CFL, Courant et al., (1928)) over the period of interest (Supplementary Figure 3). We use the MFS horizontal velocity fields of the 3$^{rd}$ and 17$^{th}$ vertical levels, which correspond to about 12 m for the shallow coastal habitat and 102 m for the neritic shelf habitat, respectively. Numerical propagules are tracked over five different drifting times (simulating different Pelagic Larval Durations): 1, 10, 20, 30 and 45 days considering successive starting times with a 10-day periodicity over years 2000 to 2010. Altogether, it represents 402 Lagrangian experiments per PLD and per vertical layer, that is 4020 numerical experiments in total (Supplementary Table 4).

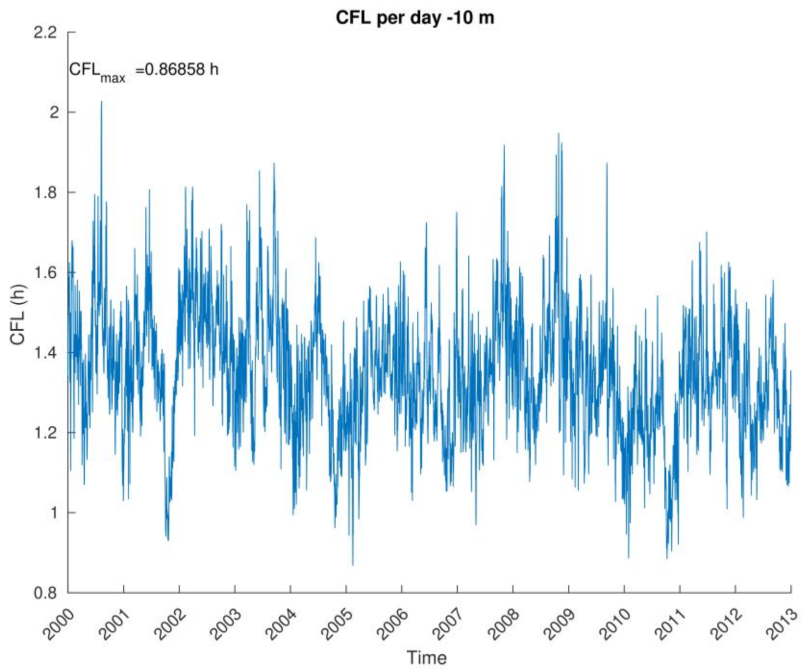


*Supplementary Figure 3:* ***CFL condition indexed for each MFS daily velocity field at 12 m deep between 2000 and 2010****. The maximum time-step value to fulfil CFL condition is 0.87 h, we use a time-step of 0.3 h in our Lagrangian experiments.*

*Supplementary Table 4:* ***Summary of the numerical experiments performed*** *using a 10-day periodicity over 2000-2010. In total, 402 Lagrangian experiments were performed for each PLD/habitat combination (i.e. five PLDs and two habitats, meaning 4020 Lagrangian experiments in total). All the 402 Lagrangian experiments were used to summarize dispersal for all year spawning species. For species characterised by seasonal spawning, we considered only Lagrangian experiments conducted during the spawning season (~ 100 experiments for each study).*

| Season | Winter | Spring | Summer | Fall | All year |
|---|---|---|---|---|---|
| Start date | 01/01 | 01/04 | 01/07 | 01/10 | 01/01 |
| End date | 31/03 | 30/06 | 30/09 | 31/12 | 31/12 |
| Nbr of Lagrangian experiment | 100 | 99 | 100 | 103 | 402 |
| Total mean nbr of particles | 12,000,000 | 12,000,000 | 12,000,000 | 12,000,000 | 48,000,000 |

## Supplementary Methods 4: Parametrization of our multi-generation dispersal models

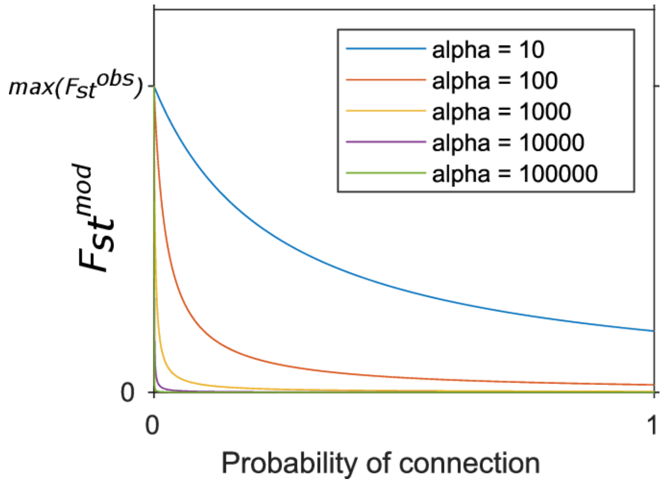


*Supplementary Figure 4:* ***Sensitivity of the reciprocal transformation applied on dispersal probabilities to obtain*** $F_{st}^{mod}$ ***with respect to the value of alpha.***

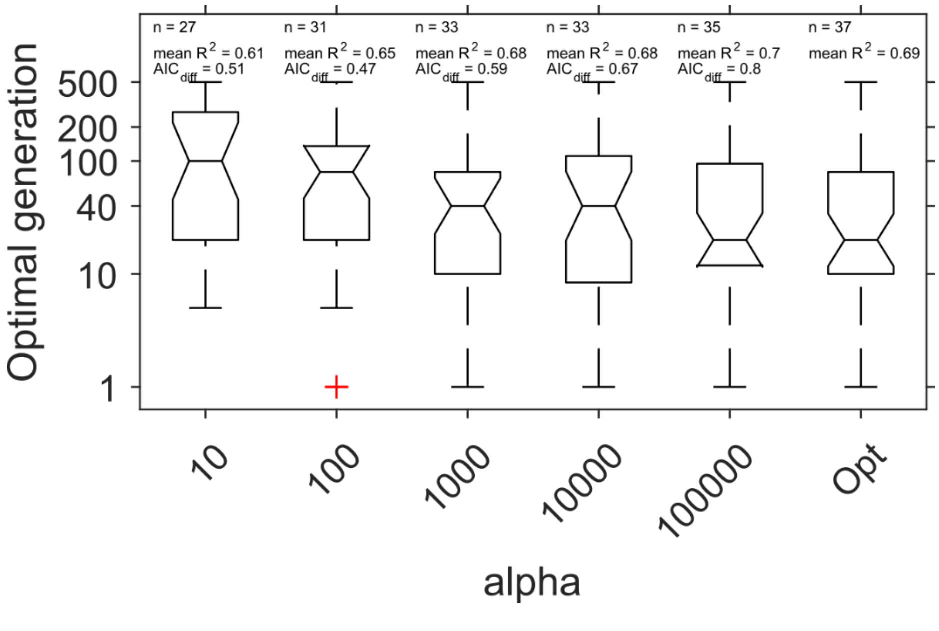


*Supplementary Figure 5:* ***Boxplot of the optimal generations obtained when varying alpha (reciprocal transformation of dispersal probabilities into*** $F_{st}^{mod}$ ***when comparing the multi-generation implicit dispersal model predictions (R², p-value and AIC) against*** $F_{st}^{obs}/(1 - F_{st}^{obs})$ ***across the whole meta-analysis.*** *n = number of studies whose model predictions of genetic observations are significant. On each box, the central mark, the bottom and top edges indicate the indicates the median, the 25th and the 75th percentiles, respectively. The whiskers extend to the most extreme data points not considered outliers, and the outliers are plotted individually using a cross symbol. Source data are provided as a Source Data file.*

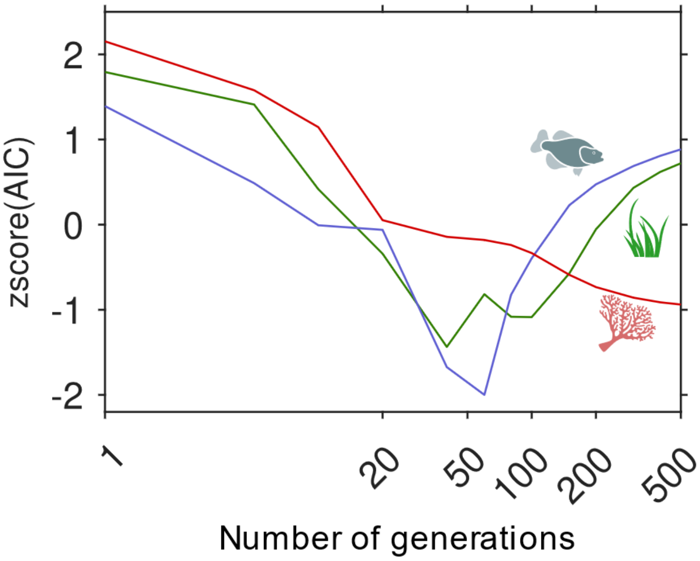


*Supplementary Figure 6:* ***Multi-generation implicit dispersal model AIC values as a function of the number of generations for three specific studies: Aurelle et al., 2011 (*Corallium rubrum* in red), Dalongeville et al., 2018 (*Mullus surmuletus* in blue) and Arnaud-Haond et al., 2007 (*Posidonia oceanica* in green).*** *Source data are provided as a Source Data file. Icons credit: © vectors market, © Agne Alesiute, © Luis Prado (changes were made on all the icons, Creative Commons BY 4.0 license).*

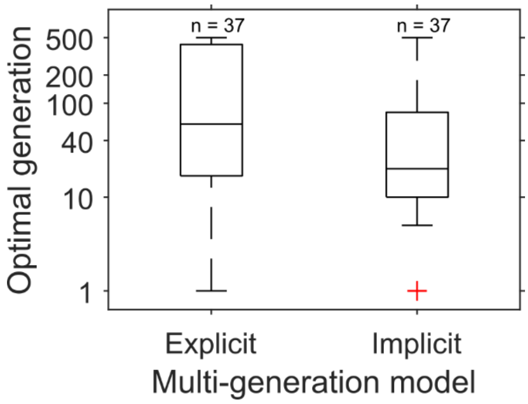


*Supplementary Figure 7:* ***Statistical distribution (boxplot) of the optimal generations for significant predictions of $F_{st}^{obs}/(1-F_{st}^{obs})$ across the meta-analysis, as obtained using the multi-generation explicit and implicit dispersal models.*** *n = number of studies whose model predictions of genetic observations are significant. On each box, the central mark, the bottom and top edges indicate the indicates the median, the 25th and the 75th percentiles, respectively. The whiskers extend to the most extreme data points not considered outliers, and the outliers are plotted individually using a cross symbol. Source data are provided as a Source Data file.*

# Supplementary Methods 5: Screening for the best models predicting observed genetic differentiation

*Supplementary Table 5:* ***Accuracy of the Isolation-by-Euclidian Distance model in explaining observed genetic structure across the meta-analysis.*** *Mantel r and MLPE linear mixed model R² between Euclidian distance and* $F_{st}^{obs}/(1-F_{st}^{obs})$ *and theirs associated p-value are indexed for all the studies. Note that (i) Weber et al., 2015 use SNPs marker ([1]) and mtDNA marker ([2]), (ii) Carrera et al., 2019 considers all the loci ([3]) and only the Mediterranean outliers loci ([4]), and (iii) Marzouk et al., 2017 use SNPs marker ([5]) and mtDNA marker ([6]). Significant studies (p-values*) are highlighted in grey. Source data are provided as a Source Data file.*

| Species | Study | Mantel *r* | Mantel p-value | MLPE R² | MLPE p-value |
|---|---|---|---|---|---|
| *Cystoseira amentacea* | Susini et al., 2007 | 0,78 | 0,0417 | 0,85 | 0,0148 |
| *Astroides calycularis* | Casado-Amezua et al., 2012 | 0,72 | 0,001 | 0,84 | 0 |
| *Corallium rubrum* | Aurelle et al., 2011 | 0,42 | 0,001 | 0,43 | 0 |
| *Corallium rubrum* | Costantini et al., 2013 | 0,54 | 0,1 | 0,31 | 0,0653 |
| *Eunicella cavolinii* | Masmoudi et al., 2016 | 0,76 | 0,001 | 0,58 | 0 |
| *Leptopsammia pruvoti* | Boscari et al., 2019 | 0,22 | 0,125 | 0,09 | 0,1201 |
| *Botryllus schlosseri* | Reem et al., 2017 | 0,36 | 0,099 | 0,17 | 0,0056 |
| *Halocynthia papillosa* | Villamor et al., 2014 | 0,87 | 0,125 | 1 | 0 |
| *Microcosmus squamiger* | Ordóñez et al., 2013 | -0,32 | 0,8972 | 0,11 | 0,1982 |
| *Pycnoclavella communis* | Pérez-Portela et al., 2007 | -0,37 | 0,7917 | 0,16 | 0,3488 |
| *Carcinus aestuarii* | Schiavina et al., 2014 | 0,46 | 0,008 | 0,93 | 0,5072 |
| *Melicertus kerathurus* | Arculeo et al., 2010 | -0,04 | 0,523 | 0 | 0,8016 |
| *Melicertus kerathurus* | Zitari-Chatti et al., 2007 | 0,11 | 0,219 | 0,05 | 0,4783 |
| *Pachygrapsus marmoratus* | Fratini et al., 2013 | 0,44 | 0,002 | 0,2 | 0,0132 |
| *Palinurus elephas* | Palero et al., 2011 | 0,24 | 0,3167 | 0,06 | 0,4472 |
| *Spongia officinalis* | Dailianis et al., 2011 | 0,72 | 0,005 | 0,68 | 0 |
| *Astropecten aranciacus* | Zulliger et al., 2009 | 0,44 | 0,014 | 0,2 | 0,0327 |
| *Holothuria mammata* | Borrero-Pérez et al., 2011 | 0,96 | 0,125 | 0,97 | 0,0001 |
| *Ophioderma longicauda* | Weber et al., 2015[1] | -0,16 | 0,777 | 0,56 | 0,8773 |
| *Ophioderma longicauda* | Weber et al., 2015[2] | -0,22 | 0,871 | 0,12 | 0,1357 |

| Species | Study | Mantel *r* | Mantel p-value | MLPE $R^2$ | MLPE p-value |
|---|---|---|---|---|---|
| *Paracentrotus lividus* | Penant et al., 2013 | -0,33 | 0,933 | 0,56 | 0,0001 |
| *Paracentrotus lividus* | Paterno et al., 2017 | 0,66 | 0,03 | 0,85 | 0,0397 |
| *Paracentrotus lividus* | Carreras et al., 2020[3] | 0,52 | 0,001 | 0,81 | 0 |
| *Paracentrotus lividus* | Carreras et al., 2020[4] | 0,56 | 0,006 | 0,81 | 0 |
| *Apogon imberbis* | Muths et al., 2015 | -0,17 | 0,625 | 0,61 | 0,9141 |
| *Coris julis* | Fruciano et al., 2011 | 0,61 | 0,044 | 0,98 | 0,7393 |
| *Diplodus sargus* | González-Wangüemert et al., 2010 | 0,26 | 0,175 | 0,08 | 0,3955 |
| *Diplodus vulgaris* | Kaouèche et al., 2013 | 0,22 | 0,2014 | 0,05 | 0,391 |
| *Epinephelus marginatus* | Schunter et al., 2011 | 0,22 | 0,173 | 0,48 | 0,0331 |
| *Lithognathus mormyrus* | Hammami et al., 2007 | 0,68 | 0,1667 | 0,51 | 0,0515 |
| *Merluccius merluccius* | Milano et al., 2014 | 0,55 | 0,001 | 0,37 | 0 |
| *Mugil cephalus* | Durand et al., 2013 | 0,29 | 0,05 | 0,14 | 0,0144 |
| *Mullus barbartus* | Maggio et al., 2009 | 0,12 | 0,215 | 0,24 | 0,0714 |
| *Mullus surmuletus* | Galarza et al., 2009 | -0,04 | 0,4514 | 0,15 | 0,9186 |
| *Mullus surmuletus* | Dalongeville et al., 2018 | 0,21 | 0,001 | 0,61 | 0,0199 |
| *Oblada melanura* | Gkafas et al., 2013 | 0,04 | 0,382 | 0 | 0,8142 |
| *Oblada melanura* | Calò et al., 2016 | 0,2 | 0,118 | 0,23 | 0,1709 |
| *Pagellus erythrinus* | Fassatoui et al., 2009 | 0,35 | 0,1792 | 0,13 | 0,1659 |
| *Serranus cabrilla* | Schunter et al., 2011 | 0,33 | 0,07 | 0,75 | 0 |
| *Solea solea* | Bahri-Sfar et al., 2011 | 0,57 | 0,009 | 0,65 | 0 |
| *Solea solea* | Garoia et al., 2007 | 0,99 | 0,0417 | 0,98 | 0 |
| *Sparus aurata* | Franchini et al., 2012 | 0,15 | 0,195 | 0,23 | 0,111 |
| *Symphodus tinca* | Carreras et al., 2017 | 0,37 | 0,175 | 0,36 | 0,4087 |
| *Cerastoderma edule* | Sromek et al., 2019 | 0,71 | 0,003 | 0,89 | 0 |
| *Chiton olivaceus* | Villamor et al., 2014 | 0,78 | 0,2083 | 0,65 | 0,0175 |
| *Hexaplex trunculus* | Villamor et al., 2014 | 0,82 | 0,125 | 0,71 | 0,0396 |
| *Hexaplex trunculus* | Marzouk et al., 2017[5] | 0,17 | 0,154 | 0,48 | 0,5448 |
| *Hexaplex trunculus* | Marzouk et al., 2017[6] | 0,2 | 0,111 | 0,52 | 0,6864 |

| **Species** | **Study** | **Mantel *r*** | **Mantel p-value** | **MLPE $R^2$** | **MLPE p-value** |
|---|---|---|---|---|---|
| *Mytilus galloprovincialis* | Diz and Presa, 2008 | 0,5 | 0,009 | 0,3 | 0,0064 |
| *Ostrea edulis* | Launey et al., 2002 | 0,4 | 0,2167 | 0,83 | 0,0097 |
| *Patella caerulea* | Villamor et al., 2014 | 0,37 | 0,1 | 0,39 | 0,0992 |
| *Patella rustica* | Sá-Pinto et al., 2012 | 0,05 | 0,3736 | 0,18 | 0,7239 |
| *Patella ulyssiponensis* | Sá-Pinto et al., 2012 | 0,39 | 0,0667 | 0,16 | 0,201 |
| *Phorcus turbinatus* | Villamor et al., 2014 | 0,84 | 0,2917 | 1 | 0,0001 |
| *Ruditapes decussatus* | Gharbi et al., 2011 | 0,42 | 0,006 | 0,18 | 0,0048 |
| *Spondylus spinosus* | Shabtay et al., 2014 | -0,05 | 0,5167 | 0,5 | 0,7273 |
| *Cymodocea nodosa* | Alberto et al., 2008 | 0,37 | 0,014 | 0,36 | 0,0002 |
| *Posidonia oceanica* | Arnaud-Haond et al., 2007 | 0,36 | 0,001 | 0,25 | 0 |

*Supplementary Table 6:* ***Accuracy of the Isolation-by-sea least cost Distance model in explaining observed genetic structure across the meta-analysis.*** *Mantel r and MLPE linear mixed model R² between sea least-cost distance and $F_{st}^{obs}/(1-F_{st}^{obs})$ and theirs associated p-value are indexed for all the studies. Note that (i) Weber et al., 2015 use SNPs marker ([1]) and mtDNA marker ([2]), (ii) Carrera et al., 2019 considers all the loci ([3]) and only the Mediterranean outliers loci ([4]), and (iii) Marzouk et al., 2017 use SNPs marker ([5]) and mtDNA marker ([6]). Significant studies (p-values*) are highlighted in grey. Source data are provided as a Source Data file.*

| Species | Study | Mantel *r* | Mantel p-value | MLPE R² | MLPE p-value |
|---|---|---|---|---|---|
| *Cystoseira amentacea* | Susini et al., 2007 | 0,78 | 0,0417 | 0,85 | 0,0148 |
| *Astroides calycularis* | Casado-Amezua et al., 2012 | 0,72 | 0,001 | 0,84 | 0 |
| *Corallium rubrum* | Aurelle et al., 2011 | 0,42 | 0,001 | 0,43 | 0 |
| *Corallium rubrum* | Costantini et al., 2013 | 0,54 | 0,1 | 0,31 | 0,0653 |
| *Eunicella cavolinii* | Masmoudi et al., 2016 | 0,76 | 0,001 | 0,58 | 0 |
| *Leptopsammia pruvoti* | Boscari et al., 2019 | 0,22 | 0,125 | 0,09 | 0,1201 |
| *Botryllus schlosseri* | Reem et al., 2017 | 0,36 | 0,099 | 0,17 | 0,0056 |
| *Halocynthia papillosa* | Villamor et al., 2014 | 0,87 | 0,125 | 1 | 0 |
| *Microcosmus squamiger* | Ordóñez et al., 2013 | -0,32 | 0,8972 | 0,11 | 0,1982 |
| *Pycnoclavella communis* | Pérez-Portela et al., 2007 | -0,37 | 0,7917 | 0,16 | 0,3488 |
| *Carcinus aestuarii* | Schiavina et al., 2014 | 0,46 | 0,008 | 0,93 | 0,5072 |
| *Melicertus kerathurus* | Arculeo et al., 2010 | -0,04 | 0,523 | 0 | 0,8016 |
| *Melicertus kerathurus* | Zitari-Chatti et al., 2007 | 0,11 | 0,219 | 0,05 | 0,4783 |
| *Pachygrapsus marmoratus* | Fratini et al., 2013 | 0,44 | 0,002 | 0,2 | 0,0132 |
| *Palinurus elephas* | Palero et al., 2011 | 0,24 | 0,3167 | 0,06 | 0,4472 |
| *Spongia officinalis* | Dailianis et al., 2011 | 0,72 | 0,005 | 0,68 | 0 |
| *Astropecten aranciacus* | Zulliger et al., 2009 | 0,44 | 0,014 | 0,2 | 0,0327 |
| *Holothuria mammata* | Borrero-Pérez et al., 2011 | 0,96 | 0,125 | 0,97 | 0,0001 |
| *Ophioderma longicauda* | Weber et al., 2015[1] | -0,16 | 0,777 | 0,56 | 0,8773 |
| *Ophioderma longicauda* | Weber et al., 2015[2] | -0,22 | 0,871 | 0,12 | 0,1357 |
| *Paracentrotus lividus* | Penant et al., 2013 | -0,33 | 0,933 | 0,56 | 0,0001 |
| *Paracentrotus lividus* | Paterno et al., 2017 | 0,66 | 0,03 | 0,85 | 0,0397 |
| *Paracentrotus lividus* | Carreras et al., 2020[3] | 0,52 | 0,001 | 0,81 | 0 |
| *Paracentrotus lividus* | Carreras et al., 2020[4] | 0,56 | 0,006 | 0,81 | 0 |

| Species | Study | Mantel *r* | Mantel p-value | MLPE $R^2$ | MLPE p-value |
|---|---|---|---|---|---|
| *Apogon imberbis* | Muths et al., 2015 | -0,17 | 0,625 | 0,61 | 0,9141 |
| *Coris julis* | Fruciano et al., 2011 | 0,61 | 0,044 | 0,98 | 0,7393 |
| *Diplodus sargus* | González-Wangüemert et al., 2010 | 0,26 | 0,175 | 0,08 | 0,3955 |
| *Diplodus vulgaris* | Kaouèche et al., 2013 | 0,22 | 0,2014 | 0,05 | 0,391 |
| *Epinephelus marginatus* | Schunter et al., 2011 | 0,22 | 0,173 | 0,48 | 0,0331 |
| *Lithognathus mormyrus* | Hammami et al., 2007 | 0,68 | 0,1667 | 0,51 | 0,0515 |
| *Merluccius merluccius* | Milano et al., 2014 | 0,55 | 0,001 | 0,37 | 0 |
| *Mugil cephalus* | Durand et al., 2013 | 0,29 | 0,05 | 0,14 | 0,0144 |
| *Mullus barbartus* | Maggio et al., 2009 | 0,12 | 0,215 | 0,24 | 0,0714 |
| *Mullus surmuletus* | Galarza et al., 2009 | -0,04 | 0,4514 | 0,15 | 0,9186 |
| *Mullus surmuletus* | Dalongeville et al., 2018 | 0,21 | 0,001 | 0,61 | 0,0199 |
| *Oblada melanura* | Gkafas et al., 2013 | 0,04 | 0,382 | 0 | 0,8142 |
| *Oblada melanura* | Calò et al., 2016 | 0,2 | 0,118 | 0,23 | 0,1709 |
| *Pagellus erythrinus* | Fassatoui et al., 2009 | 0,35 | 0,1792 | 0,13 | 0,1659 |
| *Serranus cabrilla* | Schunter et al., 2011 | 0,33 | 0,07 | 0,75 | 0 |
| *Solea solea* | Bahri-Sfar et al., 2011 | 0,57 | 0,009 | 0,65 | 0 |
| *Solea solea* | Garoia et al., 2007 | 0,99 | 0,0417 | 0,98 | 0 |
| *Sparus aurata* | Franchini et al., 2012 | 0,15 | 0,195 | 0,23 | 0,111 |
| *Symphodus tinca* | Carreras et al., 2017 | 0,37 | 0,175 | 0,36 | 0,4087 |
| *Cerastoderma edule* | Sromek et al., 2019 | 0,71 | 0,003 | 0,89 | 0 |
| *Chiton olivaceus* | Villamor et al., 2014 | 0,78 | 0,2083 | 0,65 | 0,0175 |
| *Hexaplex trunculus* | Villamor et al., 2014 | 0,82 | 0,125 | 0,71 | 0,0396 |
| *Hexaplex trunculus* | Marzouk et al., 2017[5] | 0,17 | 0,154 | 0,48 | 0,5448 |
| *Hexaplex trunculus* | Marzouk et al., 2017[6] | 0,2 | 0,111 | 0,52 | 0,6864 |
| *Mytilus galloprovincialis* | Diz and Presa, 2008 | 0,5 | 0,009 | 0,3 | 0,0064 |
| *Ostrea edulis* | Launey et al., 2002 | 0,4 | 0,2167 | 0,83 | 0,0097 |
| *Patella caerulea* | Villamor et al., 2014 | 0,37 | 0,1 | 0,39 | 0,0992 |
| *Patella rustica* | Sá-Pinto et al., 2012 | 0,05 | 0,3736 | 0,18 | 0,7239 |

| Species | Study | Mantel *r* | Mantel p-value | MLPE $R^2$ | MLPE p-value |
|---|---|---|---|---|---|
| *Patella ulyssiponensis* | Sá-Pinto et al., 2012 | 0,39 | 0,0667 | 0,16 | 0,201 |
| *Phorcus turbinatus* | Villamor et al., 2014 | 0,84 | 0,2917 | 1 | 0,0001 |
| *Ruditapes decussatus* | Gharbi et al., 2011 | 0,42 | 0,006 | 0,18 | 0,0048 |
| *Spondylus spinosus* | Shabtay et al., 2014 | -0,05 | 0,5167 | 0,5 | 0,7273 |
| *Cymodocea nodosa* | Alberto et al., 2008 | 0,37 | 0,014 | 0,36 | 0,0002 |
| *Posidonia oceanica* | Arnaud-Haond et al., 2007 | 0,36 | 0,001 | 0,25 | 0 |

*Supplementary Table 7:* ***Accuracy of our single-generation explicit dispersal model in explaining observed genetic structure across the meta-analysis.*** *Mantel r and MLPE linear mixed model R² between $F_{st}^{mod}$ and $F_{st}^{obs}/(1-F_{st}^{obs})$ and theirs associated p-value are indexed for all the studies. The "opt alpha" stands for the optimal alpha value of the reciprocal transformation that minimizes the AIC estimator (see Supplementary Figure 5). Note that (i) Weber et al., 2015 use SNPs marker ([1]) and mtDNA marker ([2]), (ii) Carrera et al., 2019 considers all the loci ([3]) and only the Mediterranean outliers loci ([4]), and (iii) Marzouk et al., 2017 use SNPs marker ([5]) and mtDNA marker ([6]). Significant studies (p-values*) are highlighted in grey. Source data are provided as a Source Data file.*

| Species | Study | Mantel *r* | Mantel p-value | Mantel opt alpha | MLPE R² | MLPE p-value | MLPE opt alpha |
|---|---|---|---|---|---|---|---|
| *Cystoseira amentacea* | Susini et al., 2007 | 0,74 | 0,1667 | 10 | 0,59 | 0,0304 | 10 |
| *Astroides calycularis* | Casado-Amezua et al., 2012 | 0,44 | 0,001 | 10000 | 0,66 | 0 | 1000 |
| *Corallium rubrum* | Aurelle et al., 2011 | 0,3 | 0,001 | 1000 | 0,4 | 0 | 100000 |
| *Corallium rubrum* | Costantini et al., 2013 | 0,49 | 0,1 | 10 | 0,26 | 0,1029 | 10 |
| *Eunicella cavolinii* | Masmoudi et al., 2016 | 0,68 | 0,001 | 100000 | 0,46 | 0 | 100000 |
| *Leptopsammia pruvoti* | Boscari et al., 2019 | 0,27 | 0,012 | 100000 | 0,11 | 0,0642 | 100000 |
| *Botryllus schlosseri* | Reem et al., 2017 | NaN | NaN | NaN | NaN | NaN | NaN |
| *Halocynthia papillosa* | Villamor et al., 2014 | NaN | NaN | NaN | NaN | NaN | NaN |
| *Microcosmus squamiger* | Ordóñez et al., 2013 | -0,07 | 0,6667 | 10 | 0 | 0,7982 | 10 |
| *Pycnoclavella communis* | Pérez-Portela et al., 2007 | -0,41 | 0,8333 | 10 | 0,2 | 0,2879 | 10 |
| *Carcinus aestuarii* | Schiavina et al., 2014 | 0,37 | 0,028 | 100000 | 0,94 | 0,3879 | 100 |
| *Melicertus kerathurus* | Arculeo et al., 2010 | 0,04 | 0,597 | 10 | 0,01 | 0,5952 | 100000 |
| *Melicertus kerathurus* | Zitari-Chatti et al., 2007 | 0,13 | 0,237 | 100000 | 0,06 | 0,4233 | 100000 |
| *Pachygrapsus marmoratus* | Fratini et al., 2013 | 0,01 | 0,52 | 10 | 0,01 | 0,6935 | 100000 |
| *Palinurus elephas* | Palero et al., 2011 | NaN | NaN | NaN | NaN | NaN | NaN |
| *Spongia officinalis* | Dailianis et al., 2011 | 0,25 | 0,068 | 100000 | 0,31 | 0,0364 | 100000 |
| *Astropecten aranciacus* | Zulliger et al., 2009 | NaN | NaN | NaN | NaN | NaN | NaN |
| *Holothuria mammata* | Borrero-Pérez et al., 2011 | 0,44 | 0,3333 | 10 | 0,22 | 0,2543 | 10 |
| *Ophioderma longicauda* | Weber et al., 2015[1] | -0,06 | 0,662 | 10 | 0,56 | 0,1217 | 100000 |
| *Ophioderma longicauda* | Weber et al., 2015[2] | -0,16 | 0,782 | 100000 | 0,11 | 0,1647 | 10 |
| *Paracentrotus lividus* | Penant et al., 2013 | -0,24 | 0,906 | 10 | 0,46 | 0,0024 | 100000 |
| *Paracentrotus lividus* | Paterno et al., 2017 | 0,43 | 0,038 | 100000 | 0,88 | 0,0971 | 1000 |
| *Paracentrotus lividus* | Carreras et al., 2020[3] | 0,35 | 0,038 | 10 | 0,68 | 0,0005 | 1000 |

| Species | Study | Mantel *r* | Mantel p-value | Mantel opt alpha | MLPE R² | MLPE p-value | MLPE opt alpha |
|---|---|---|---|---|---|---|---|
| *Paracentrotus lividus* | Carreras et al., 2020[4] | 0,51 | 0,04 | 100000 | 0,73 | 0,0001 | 100000 |
| *Apogon imberbis* | Muths et al., 2015 | -0,65 | 1 | 10 | 0,47 | 0,2004 | 10 |
| *Coris julis* | Fruciano et al., 2011 | 0,12 | 0,113 | 100000 | 0,98 | 0,7344 | 100000 |
| *Diplodus sargus* | González-Wangüemert et al., 2010 | 0,11 | 0,6 | 10 | 0,01 | 0,7217 | 10 |
| *Diplodus vulgaris* | Kaouèche et al., 2013 | 0,21 | 0,2417 | 100000 | 0,06 | 0,3602 | 100000 |
| *Epinephelus marginatus* | Schunter et al., 2011 | 0,16 | 0,16 | 10 | 0,45 | 0,1263 | 10000 |
| *Lithognathus mormyrus* | Hammami et al., 2007 | 0,69 | 0,2083 | 100000 | 0,53 | 0,0476 | 100000 |
| *Merluccius merluccius* | Milano et al., 2014 | 0,18 | 0,022 | 100000 | 0,14 | 0,069 | 100000 |
| *Mugil cephalus* | Durand et al., 2013 | 0,16 | 0,074 | 100000 | 0,04 | 0,1277 | 100000 |
| *Mullus barbartus* | Maggio et al., 2009 | 0,08 | 0,201 | 10 | 0,15 | 0,2698 | 10 |
| *Mullus surmuletus* | Galarza et al., 2009 | 0,29 | 0,0806 | 1000 | 0,2 | 0,2659 | 1000 |
| *Mullus surmuletus* | Dalongeville et al., 2018 | 0,01 | 0,379 | 100000 | 0,62 | 0,3775 | 10 |
| *Oblada melanura* | Gkafas et al., 2013 | -0,07 | 0,589 | 10 | 0,04 | 0,2833 | 10000 |
| *Oblada melanura* | Calò et al., 2016 | 0,21 | 0,138 | 10000 | 0,61 | 0,1171 | 10000 |
| *Pagellus erythrinus* | Fassatoui et al., 2009 | 0,46 | 0,0389 | 100000 | 0,23 | 0,0534 | 100000 |
| *Serranus cabrilla* | Schunter et al., 2011 | 0,2 | 0,036 | 100 | 0,81 | 0 | 10 |
| *Solea solea* | Bahri-Sfar et al., 2011 | 0,38 | 0,003 | 100000 | 0,14 | 0,0089 | 100000 |
| *Solea solea* | Garoia et al., 2007 | NaN | NaN | NaN | NaN | NaN | NaN |
| *Sparus aurata* | Franchini et al., 2012 | 0,32 | 0,044 | 100000 | 0,38 | 0,0004 | 100000 |
| *Symphodus tinca* | Carreras et al., 2017 | 0,32 | 0,1139 | 100000 | 0,41 | 0,3075 | 100000 |
| *Cerastoderma edule* | Sromek et al., 2019 | 0,36 | 0,048 | 1000 | 0,13 | 0,0897 | 10 |
| *Chiton olivaceus* | Villamor et al., 2014 | 0,44 | 0,3333 | 10 | 0,23 | 0,2523 | 10 |
| *Hexaplex trunculus* | Villamor et al., 2014 | NaN | NaN | NaN | NaN | NaN | NaN |
| *Hexaplex trunculus* | Marzouk et al., 2017[5] | 0 | 0,545 | 100000 | 0,5 | 0,2692 | 100000 |
| *Hexaplex trunculus* | Marzouk et al., 2017[6] | 0,05 | 0,328 | 100000 | 0,52 | 0,4083 | 10 |
| *Mytilus galloprovincialis* | Diz and Presa, 2008 | 0,41 | 0,006 | 1000 | 0,28 | 0,0245 | 1000 |
| *Ostrea edulis* | Launey et al., 2002 | 0,41 | 0,2 | 10 | 0,55 | 0,0723 | 10000 |

| **Species** | **Study** | **Mantel *r*** | **Mantel p-value** | **Mantel opt alpha** | **MLPE $R^2$** | **MLPE p-value** | **MLPE opt alpha** |
|---|---|---|---|---|---|---|---|
| *Patella caerulea* | Villamor et al., 2014 | 0,47 | 0,0444 | 1000 | 0,23 | 0,055 | 1000 |
| *Patella rustica* | Sá-Pinto et al., 2012 | 0,31 | 0,0667 | 10 | 0,18 | 0,2184 | 10 |
| *Patella ulyssiponensis* | Sá-Pinto et al., 2012 | 0,25 | 0,2 | 10 | 0,07 | 0,4257 | 10 |
| *Phorcus turbinatus* | Villamor et al., 2014 | 0,91 | 0,3333 | 100000 | 1 | 0 | 100000 |
| *Ruditapes decussatus* | Gharbi et al., 2011 | 0,17 | 0,089 | 1000 | 0,16 | 0,3376 | 100000 |
| *Spondylus spinosus* | Shabtay et al., 2014 | 0,24 | 0,4333 | 10 | 0,46 | 0,6774 | 10 |
| *Cymodocea nodosa* | Alberto et al., 2008 | 0,44 | 0,002 | 100000 | 0,36 | 0 | 100000 |
| *Posidonia oceanica* | Arnaud-Haond et al., 2007 | 0,3 | 0,001 | 100000 | 0,19 | 0 | 100000 |

*Supplementary Table 8:* ***Accuracy of our multi-generation explicit dispersal model in explaining observed genetic structure across the meta-analysis.*** *Mantel r and MLPE linear mixed model R² between $F_{st}^{mod}$ and $F_{st}^{obs}/(1-F_{st}^{obs})$ and theirs associated p-value are indexed for all the studies. The "opt alpha" stands for the alpha value of the reciprocal transformation that minimizes the AIC estimator (see Supplementary Figure 5). The "opt M" stands for the number of generations that minimizes the AIC estimator (see Supplementary Figure 6). Note that (i) Weber et al., 2015 use SNPs marker ([1]) and mtDNA marker ([2]), (ii) Carrera et al., 2019 considers all the loci ([3]) and only the Mediterranean outliers loci ([4]), and (iii) Marzouk et al., 2017 use SNPs marker ([5]) and mtDNA marker ([6]). Significant studies (p-values*) are highlighted in grey. Source data are provided as a Source Data file.*

| **Species** | **Study** | **Mantel *r*** | **Mantel p-value** | **Mantel opt M** | **Mantel opt alpha** | **MLPE R²** | **MLPE p-value** | **MLPE opt M** | **MLPE opt alpha** |
|---|---|---|---|---|---|---|---|---|---|
| *Cystoseira amentacea* | Susini et al., 2007 | 0,77 | 0,1667 | 1 | 10 | 0,59 | 0,0304 | 1 | 10 |
| *Astroides calycularis* | Casado-Amezua et al., 2012 | 0,57 | 0,001 | 300 | 100000 | 0,66 | 0 | 300 | 100000 |
| *Corallium rubrum* | Aurelle et al., 2011 | 0,71 | 0,001 | 40 | 100000 | 0,5 | 0 | 40 | 100000 |
| *Corallium rubrum* | Costantini et al., 2013 | 0,5 | 0,1 | 1 | 10 | 0,26 | 0,1029 | 1 | 10 |
| *Eunicella cavolinii* | Masmoudi et al., 2016 | 0,88 | 0,001 | 300 | 1000 | 0,78 | 0 | 300 | 1000 |
| *Leptopsammia pruvoti* | Boscari et al., 2019 | 0,59 | 0,002 | 400 | 100000 | 0,35 | 0 | 400 | 100000 |
| *Botryllus schlosseri* | Reem et al., 2017 | 0,36 | 0,006 | 500 | 100000 | 0,11 | 0,0118 | 500 | 100000 |
| *Halocynthia papillosa* | Villamor et al., 2014 | 0,9 | 0,125 | 200 | 10 | 1 | 0 | 200 | 100000 |
| *Microcosmus squamiger* | Ordóñez et al., 2013 | -0,07 | 0,6667 | 1 | 10 | 0 | 0,7982 | 1 | 10 |
| *Pycnoclavella communis* | Pérez-Portela et al., 2007 | -0,41 | 0,75 | 500 | 100000 | 0,2 | 0,2879 | 500 | 10 |
| *Carcinus aestuarii* | Schiavina et al., 2014 | 0,99 | 0,017 | 40 | 100000 | 0,97 | 0 | 40 | 100000 |
| *Melicertus kerathurus* | Arculeo et al., 2010 | 0,03 | 0,479 | 5 | 10 | 0,06 | 0,1444 | 5 | 100000 |
| *Melicertus kerathurus* | Zitari-Chatti et al., 2007 | 0,14 | 0,206 | 1 | 100000 | 0,06 | 0,4233 | 1 | 100000 |
| *Pachygrapsus marmoratus* | Fratini et al., 2013 | 0,04 | 0,327 | 500 | 10 | 0,05 | 0,2558 | 500 | 100000 |
| *Palinurus elephas* | Palero et al., 2011 | 0,29 | 0,2333 | 40 | 100000 | 0,36 | 0,0414 | 40 | 10 |

| **Species** | **Study** | **Mantel *r*** | **Mantel p-value** | **Mantel opt M** | **Mantel opt alpha** | **MLPE R²** | **MLPE p-value** | **MLPE opt M** | **MLPE opt alpha** |
|---|---|---|---|---|---|---|---|---|---|
| *Spongia officinalis* | Dailianis et al., 2011 | 0,62 | 0,009 | 500 | 100000 | 0,74 | 0 | 500 | 10000 |
| *Astropecten aranciacus* | Zulliger et al., 2009 | 0,34 | 0,075 | 5 | 10 | 0,12 | 0,1038 | 5 | 10 |
| *Holothuria mammata* | Borrero-Pérez et al., 2011 | 1 | 0,0417 | 100 | 100000 | 0,99 | 0 | 100 | 100000 |
| *Ophioderma longicauda* | Weber et al., 2015[1] | 0,05 | 0,415 | 500 | 100000 | 0,56 | 0,1217 | 500 | 100000 |
| *Ophioderma longicauda* | Weber et al., 2015[2] | 0,01 | 0,449 | 150 | 100000 | 0,11 | 0,1647 | 150 | 10 |
| *Paracentrotus lividus* | Penant et al., 2013 | 0,06 | 0,454 | 80 | 100000 | 0,49 | 0,0012 | 80 | 1000 |
| *Paracentrotus lividus* | Paterno et al., 2017 | 0,89 | 0,008 | 40 | 10000 | 0,9 | 0,0001 | 40 | 1000 |
| *Paracentrotus lividus* | Carreras et al., 2020[3] | 0,4 | 0,036 | 20 | 100000 | 0,8 | 0 | 20 | 100000 |
| *Paracentrotus lividus* | Carreras et al., 2020[4] | 0,63 | 0,008 | 5 | 100000 | 0,92 | 0 | 5 | 100000 |
| *Apogon imberbis* | Muths et al., 2015 | 0,65 | 0,25 | 5 | 10 | 0,47 | 0,2004 | 5 | 10 |
| *Coris julis* | Fruciano et al., 2011 | 0,4 | 0,049 | 80 | 100000 | 0,99 | 0,0479 | 80 | 10000 |
| *Diplodus sargus* | González-Wangüemert et al., 2010 | 0,32 | 0,2167 | 10 | 10000 | 0,44 | 0,0204 | 10 | 10 |
| *Diplodus vulgaris* | Kaouèche et al., 2013 | 0,34 | 0,1306 | 5 | 100000 | 0,13 | 0,1686 | 5 | 100000 |
| *Epinephelus marginatus* | Schunter et al., 2011 | 0,24 | 0,163 | 10 | 100000 | 0,49 | 0,0491 | 10 | 1000 |
| *Lithognathus mormyrus* | Hammami et al., 2007 | 0,84 | 0,0833 | 5 | 100000 | 0,75 | 0,0058 | 5 | 100000 |
| *Merluccius merluccius* | Milano et al., 2014 | 0,28 | 0,001 | 40 | 10 | 0,2 | 0,011 | 40 | 100000 |
| *Mugil cephalus* | Durand et al., 2013 | 0,61 | 0,002 | 20 | 100000 | 0,72 | 0 | 20 | 100000 |
| *Mullus barbartus* | Maggio et al., 2009 | 0,1 | 0,164 | 5 | 10 | 0,1 | 0,1825 | 5 | 1000 |

| Species | Study | Mantel *r* | Mantel p-value | Mantel opt M | Mantel opt alpha | MLPE $R^2$ | MLPE p-value | MLPE opt M | MLPE opt alpha |
|---|---|---|---|---|---|---|---|---|---|
| *Mullus surmuletus* | Galarza et al., 2009 | 0,34 | 0,1986 | 500 | 100 | 0,25 | 0,1794 | 500 | 10000 |
| *Mullus surmuletus* | Dalongeville et al., 2018 | 0,21 | 0,017 | 60 | 100000 | 0,67 | 0 | 60 | 100000 |
| *Oblada melanura* | Gkafas et al., 2013 | 0,01 | 0,447 | 500 | 10 | 0,04 | 0,2833 | 500 | 10000 |
| *Oblada melanura* | Calò et al., 2016 | 0,21 | 0,113 | 1 | 10000 | 0,77 | 0,0084 | 1 | 100000 |
| *Pagellus erythrinus* | Fassatoui et al., 2009 | 0,46 | 0,0389 | 1 | 100000 | 0,23 | 0,0534 | 1 | 100000 |
| *Serranus cabrilla* | Schunter et al., 2011 | 0,2 | 0,024 | 1 | 100 | 0,88 | 0 | 1 | 10 |
| *Solea solea* | Bahri-Sfar et al., 2011 | 0,9 | 0,005 | 20 | 10000 | 0,86 | 0 | 20 | 10000 |
| *Solea solea* | Garoia et al., 2007 | 0,49 | 0,1667 | 5 | 10 | 0,47 | 0,0686 | 5 | 100000 |
| *Sparus aurata* | Franchini et al., 2012 | 0,41 | 0,043 | 400 | 100 | 0,43 | 0 | 400 | 10 |
| *Symphodus tinca* | Carreras et al., 2017 | 0,35 | 0,0764 | 5 | 10 | 0,52 | 0,1206 | 5 | 100000 |
| *Cerastoderma edule* | Sromek et al., 2019 | 0,66 | 0,018 | 5 | 100000 | 0,67 | 0,0002 | 5 | 100000 |
| *Chiton olivaceus* | Villamor et al., 2014 | 0,82 | 0,1667 | 500 | 10000 | 0,71 | 0,01 | 500 | 1000 |
| *Hexaplex trunculus* | Villamor et al., 2014 | 0,94 | 0,0833 | 200 | 100000 | 0,88 | 0,0023 | 200 | 10000 |
| *Hexaplex trunculus* | Marzouk et al., 2017[5] | 0,25 | 0,093 | 150 | 10 | 0,49 | 0,0984 | 150 | 100000 |
| *Hexaplex trunculus* | Marzouk et al., 2017[6] | 0,31 | 0,048 | 300 | 10 | 0,52 | 0,1023 | 300 | 10 |
| *Mytilus galloprovincialis* | Diz and Presa, 2008 | 0,41 | 0,006 | 1 | 1000 | 0,28 | 0,0245 | 1 | 1000 |
| *Ostrea edulis* | Launey et al., 2002 | 0,71 | 0,0333 | 40 | 1000 | 0,59 | 0,0059 | 40 | 10000 |
| *Patella caerulea* | Villamor et al., 2014 | 0,91 | 0,0153 | 40 | 100000 | 0,86 | 0 | 40 | 100000 |
| *Patella rustica* | Sá-Pinto et al., 2012 | 0,34 | 0,0389 | 5 | 10 | 0,45 | 0,1975 | 5 | 100000 |
| *Patella ulyssiponensis* | Sá-Pinto et al., 2012 | 0,64 | 0,05 | 80 | 100000 | 0,88 | 0,0016 | 80 | 100000 |
| *Phorcus turbinatus* | Villamor et al., 2014 | 0,95 | 0,3333 | 40 | 100000 | 1 | 0 | 40 | 100000 |
| *Ruditapes decussatus* | Gharbi et al., 2011 | 0,41 | 0,006 | 5 | 100000 | 0,17 | 0,0073 | 5 | 100000 |
| *Spondylus spinosus* | Shabtay et al., 2014 | 0,38 | 0,1667 | 5 | 100000 | 0,45 | 0,4206 | 5 | 100000 |
| *Cymodocea nodosa* | Alberto et al., 2008 | 0,75 | 0,011 | 10 | 10000 | 0,77 | 0 | 10 | 100000 |
| *Posidonia oceanica* | Arnaud-Haond et al., 2007 | 0,58 | 0,001 | 40 | 10000 | 0,58 | 0 | 40 | 10000 |

*Supplementary Table 9:* ***Accuracy of our multi-generation implicit dispersal models in explaining observed genetic structure across the meta-analysis.*** *Mantel r and MLPE linear mixed model R² between $F_{st}^{mod}$ and $F_{st}^{obs}/(1-F_{st}^{obs})$ and theirs associated p-value are indexed for all the studies. The "opt alpha" stands for the alpha value of the reciprocal transformation that minimizes the AIC estimator (see Supplementary Figure 5). The "opt M" stands for the number of generations that minimizes the AIC estimator (see Supplementary Figure 6). Note that (i) Weber et al., 2015 use SNPs marker ([1]) and mtDNA marker ([2]), (ii) Carrera et al., 2019 considers all the loci ([3]) and only the Mediterranean outliers loci ([4]), and (iii) Marzouk et al., 2017 use SNPs marker ([5]) and mtDNA marker ([6]). Significant studies (p-values*) are highlighted in grey. Source data are provided as a Source Data file.*

| **Species** | **Study** | **Mantel *r*** | **Mantel p-value** | **Mantel opt M** | **Mantel opt alpha** | **MLPE R²** | **MLPE p-value** | **MLPE opt M** | **MLPE opt alpha** |
|---|---|---|---|---|---|---|---|---|---|
| *Cystoseira amentacea* | Susini et al., 2007 | 0,81 | 0,0833 | 1 | 100000 | 0,74 | 0,0194 | 1 | 100000 |
| *Astroides calycularis* | Casado-Amezua et al., 2012 | 0,67 | 0,005 | 500 | 100000 | 0,79 | 0 | 500 | 100000 |
| *Corallium rubrum* | Aurelle et al., 2011 | 0,71 | 0,001 | 40 | 10000 | 0,54 | 0 | 40 | 100000 |
| *Corallium rubrum* | Costantini et al., 2013 | 0,51 | 0,1 | 1 | 10 | 0,27 | 0,0928 | 1 | 10 |
| *Eunicella cavolinii* | Masmoudi et al., 2016 | 0,88 | 0,001 | 300 | 1000 | 0,78 | 0 | 300 | 10000 |
| *Leptopsammia pruvoti* | Boscari et al., 2019 | 0,6 | 0,002 | 40 | 10 | 0,4 | 0 | 40 | 100000 |
| *Botryllus schlosseri* | Reem et al., 2017 | 0,36 | 0,006 | 500 | 100000 | 0,11 | 0,0132 | 500 | 100000 |
| *Halocynthia papillosa* | Villamor et al., 2014 | 0,9 | 0,1667 | 20 | 100 | 1 | 0 | 20 | 10 |
| *Microcosmus squamiger* | Ordóñez et al., 2013 | -0,07 | 0,6667 | 1 | 10 | 0 | 0,7982 | 1 | 10 |
| *Pycnoclavella communis* | Pérez-Portela et al., 2007 | -0,27 | 0,5833 | 500 | 100000 | 0,2 | 0,2879 | 500 | 10 |
| *Carcinus aestuarii* | Schiavina et al., 2014 | 0,98 | 0,022 | 40 | 10000 | 0,97 | 0 | 40 | 10000 |
| *Melicertus kerathurus* | Arculeo et al., 2010 | 0,14 | 0,32 | 40 | 10 | 0,07 | 0,113 | 40 | 10000 |
| *Melicertus kerathurus* | Zitari-Chatti et al., 2007 | 0,19 | 0,206 | 10 | 10 | 0,04 | 0,2453 | 10 | 10 |
| *Pachygrapsus marmoratus* | Fratini et al., 2013 | 0,19 | 0,142 | 1 | 10 | 0,04 | 0,3206 | 1 | 10 |
| *Palinurus elephas* | Palero et al., 2011 | 0,26 | 0,4333 | 20 | 100000 | 0,27 | 0,0909 | 20 | 10 |

| Species | Study | Mantel *r* | Mantel p-value | Mantel opt M | Mantel opt alpha | MLPE $R^2$ | MLPE p-value | MLPE opt M | MLPE opt alpha |
|---|---|---|---|---|---|---|---|---|---|
| *Spongia officinalis* | Dailianis et al., 2011 | 0,63 | 0,006 | 500 | 10000 | 0,75 | 0 | 500 | 100000 |
| *Astropecten aranciacus* | Zulliger et al., 2009 | 0,33 | 0,043 | 10 | 100000 | 0,25 | 0,0567 | 10 | 100000 |
| *Holothuria mammata* | Borrero-Pérez et al., 2011 | 0,99 | 0,0833 | 40 | 100000 | 1 | 0 | 40 | 100000 |
| *Ophioderma longicauda* | Weber et al., 2015[1] | 0,05 | 0,307 | 500 | 100000 | 0,56 | 0,0957 | 500 | 100000 |
| *Ophioderma longicauda* | Weber et al., 2015[2] | 0,02 | 0,46 | 500 | 100000 | 0,11 | 0,1507 | 500 | 100 |
| *Paracentrotus lividus* | Penant et al., 2013 | 0,11 | 0,467 | 40 | 100000 | 0,5 | 0,0008 | 40 | 100000 |
| *Paracentrotus lividus* | Paterno et al., 2017 | 0,88 | 0,004 | 40 | 10000 | 0,81 | 0,0251 | 40 | 10000 |
| *Paracentrotus lividus* | Carreras et al., 2020[3] | 0,39 | 0,045 | 20 | 100000 | 0,8 | 0 | 20 | 100000 |
| *Paracentrotus lividus* | Carreras et al., 2020[4] | 0,6 | 0,011 | 20 | 100000 | 0,93 | 0 | 20 | 1000 |
| *Apogon imberbis* | Muths et al., 2015 | -0,65 | 1 | 1 | 10 | 0,47 | 0,2004 | 1 | 10 |
| *Coris julis* | Fruciano et al., 2011 | 0,62 | 0,05 | 400 | 10000 | 0,99 | 0,0005 | 400 | 100000 |
| *Diplodus sargus* | González-Wangüemert et al., 2010 | 0,31 | 0,1333 | 10 | 10000 | 0,11 | 0,3129 | 10 | 100000 |
| *Diplodus vulgaris* | Kaouèche et al., 2013 | 0,3 | 0,1167 | 1 | 10000 | 0,1 | 0,2328 | 1 | 1000 |
| *Epinephelus marginatus* | Schunter et al., 2011 | 0,24 | 0,137 | 40 | 10 | 0,49 | 0,0497 | 40 | 1000 |
| *Lithognathus mormyrus* | Hammami et al., 2007 | 0,71 | 0,0417 | 5 | 10 | 0,63 | 0,0218 | 5 | 100000 |
| *Merluccius merluccius* | Milano et al., 2014 | 0,3 | 0,014 | 500 | 10 | 0,2 | 0,0037 | 500 | 100000 |
| *Mugil cephalus* | Durand et al., 2013 | 0,68 | 0,02 | 20 | 100000 | 0,73 | 0 | 20 | 10000 |
| *Mullus barbartus* | Maggio et al., 2009 | 0,14 | 0,109 | 5 | 1000 | 0,17 | 0,0921 | 5 | 100 |

| Species | Study | Mantel *r* | Mantel p-value | Mantel opt M | Mantel opt alpha | MLPE $R^2$ | MLPE p-value | MLPE opt M | MLPE opt alpha |
|---|---|---|---|---|---|---|---|---|---|
| *Mullus surmuletus* | Galarza et al., 2009 | 0,24 | 0,3194 | 1 | 10 | 0,42 | 0,1082 | 1 | 100000 |
| *Mullus surmuletus* | Dalongeville et al., 2018 | 0,34 | 0,004 | 500 | 100 | 0,67 | 0 | 500 | 100000 |
| *Oblada melanura* | Gkafas et al., 2013 | 0,1 | 0,24 | 100 | 10 | 0,04 | 0,3129 | 100 | 10000 |
| *Oblada melanura* | Calò et al., 2016 | 0,3 | 0,021 | 20 | 10 | 0,75 | 0,0091 | 20 | 100000 |
| *Pagellus erythrinus* | Fassatoui et al., 2009 | 0,46 | 0,0389 | 20 | 10 | 0,65 | 0,021 | 20 | 100000 |
| *Serranus cabrilla* | Schunter et al., 2011 | 0,21 | 0,047 | 1 | 1000 | 0,88 | 0 | 1 | 10 |
| *Solea solea* | Bahri-Sfar et al., 2011 | 0,9 | 0,003 | 10 | 100000 | 0,83 | 0 | 10 | 100000 |
| *Solea solea* | Garoia et al., 2007 | 0,49 | 0,1667 | 5 | 10 | 0,49 | 0,0619 | 5 | 100000 |
| *Sparus aurata* | Franchini et al., 2012 | 0,3 | 0,046 | 10 | 10 | 0,36 | 0,0003 | 10 | 1000 |
| *Symphodus tinca* | Carreras et al., 2017 | 0,61 | 0,0194 | 20 | 100000 | 0,45 | 0,0277 | 20 | 100000 |
| *Cerastoderma edule* | Sromek et al., 2019 | 0,94 | 0,004 | 10 | 100000 | 0,96 | 0 | 10 | 10000 |
| *Chiton olivaceus* | Villamor et al., 2014 | 0,83 | 0,1667 | 150 | 10 | 0,72 | 0,0091 | 150 | 10 |
| *Hexaplex trunculus* | Villamor et al., 2014 | 0,94 | 0,0833 | 100 | 10000 | 0,88 | 0,0022 | 100 | 100000 |
| *Hexaplex trunculus* | Marzouk et al., 2017[5] | 0,26 | 0,087 | 500 | 10 | 0,48 | 0,1073 | 500 | 100 |
| *Hexaplex trunculus* | Marzouk et al., 2017[6] | 0,28 | 0,043 | 400 | 100000 | 0,52 | 0,1092 | 400 | 10 |
| *Mytilus galloprovincialis* | Diz and Presa, 2008 | 0,37 | 0,012 | 1 | 1000 | 0,43 | 0,0264 | 1 | 10 |
| *Ostrea edulis* | Launey et al., 2002 | 0,65 | 0,0333 | 150 | 10 | 0,61 | 0,0142 | 150 | 10 |
| *Patella caerulea* | Villamor et al., 2014 | 0,97 | 0,0167 | 60 | 10000 | 0,95 | 0 | 60 | 10000 |
| *Patella rustica* | Sá-Pinto et al., 2012 | 0,35 | 0,0208 | 5 | 10 | 0,44 | 0,0583 | 5 | 100000 |
| *Patella ulyssiponensis* | Sá-Pinto et al., 2012 | 0,76 | 0,025 | 100 | 100000 | 0,86 | 0,0009 | 100 | 100000 |
| *Phorcus turbinatus* | Villamor et al., 2014 | 0,99 | 0,0417 | 150 | 10 | 1 | 0 | 150 | 10 |
| *Ruditapes decussatus* | Gharbi et al., 2011 | 0,48 | 0,005 | 5 | 100000 | 0,22 | 0,0009 | 5 | 1000 |
| *Spondylus spinosus* | Shabtay et al., 2014 | 0,36 | 0,3333 | 1 | 10 | 0,45 | 0,4516 | 1 | 10 |
| *Cymodocea nodosa* | Alberto et al., 2008 | 0,8 | 0,004 | 5 | 100000 | 0,77 | 0 | 5 | 10000 |
| *Posidonia oceanica* | Arnaud-Haond et al., 2007 | 0,57 | 0,001 | 40 | 1000 | 0,58 | 0 | 40 | 100000 |

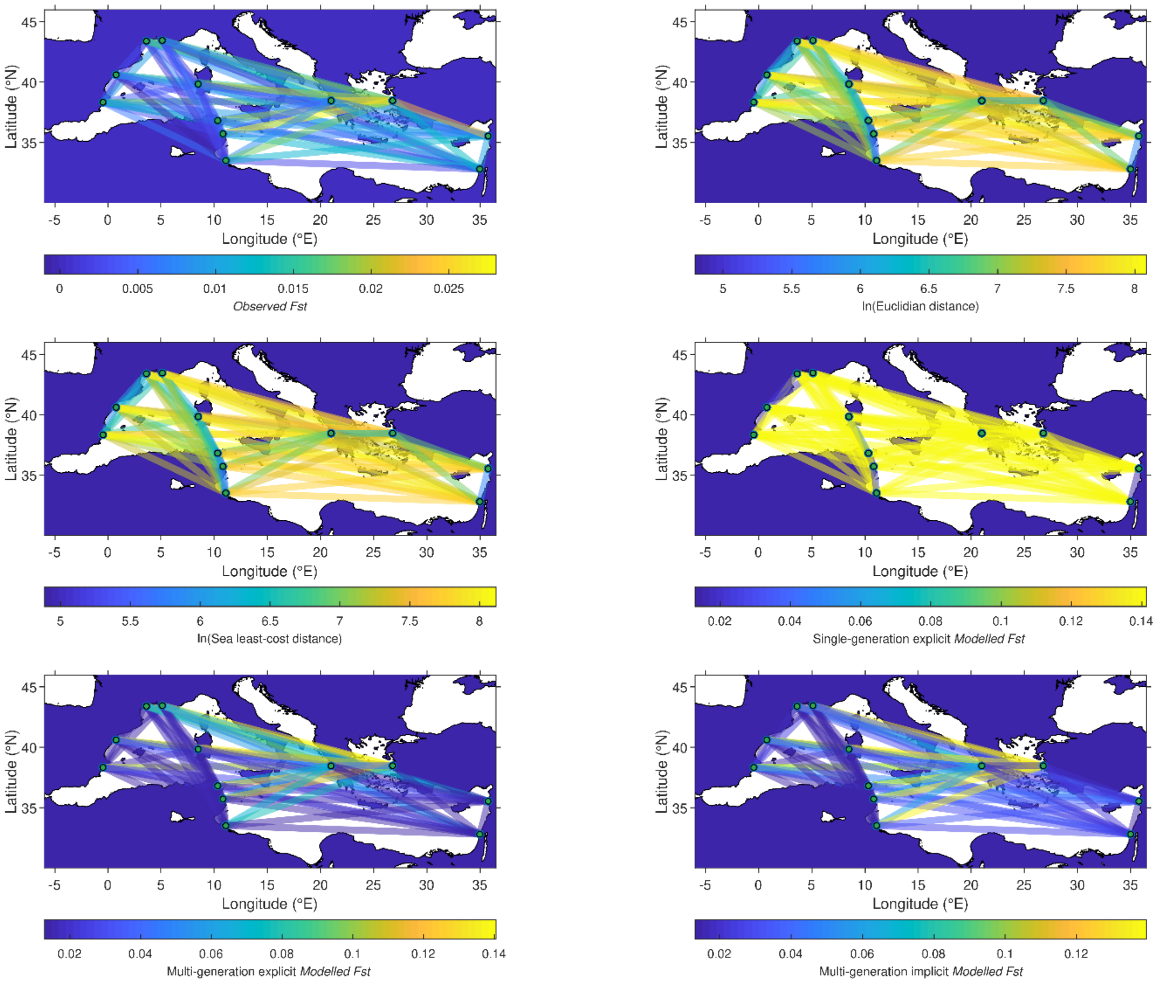


*Supplementary Figure 8:* ***Network representation of*** **observed** ***and*** **modelled** ***genetic structures*** *for* Mugil cephalus; *Durand et al., (2013).* ***a*** *$F_{st}^{obs}$ values,* ***b*** *log$_e$(Euclidian distance),* ***c*** *log$_e$(Sea least-cost distance),* ***d*** *single-generation explicit $F_{st}^{mod}$,* ***e*** *multi-generation explicit $F_{st}^{mod}$ and* ***f*** *multi-generation implicit $F_{st}^{mod}$. Similar plots made for each study of our meta-analysis can be viewed at: https://nuage.osupytheas.fr/s/3fj5TidxC4A6Kax*

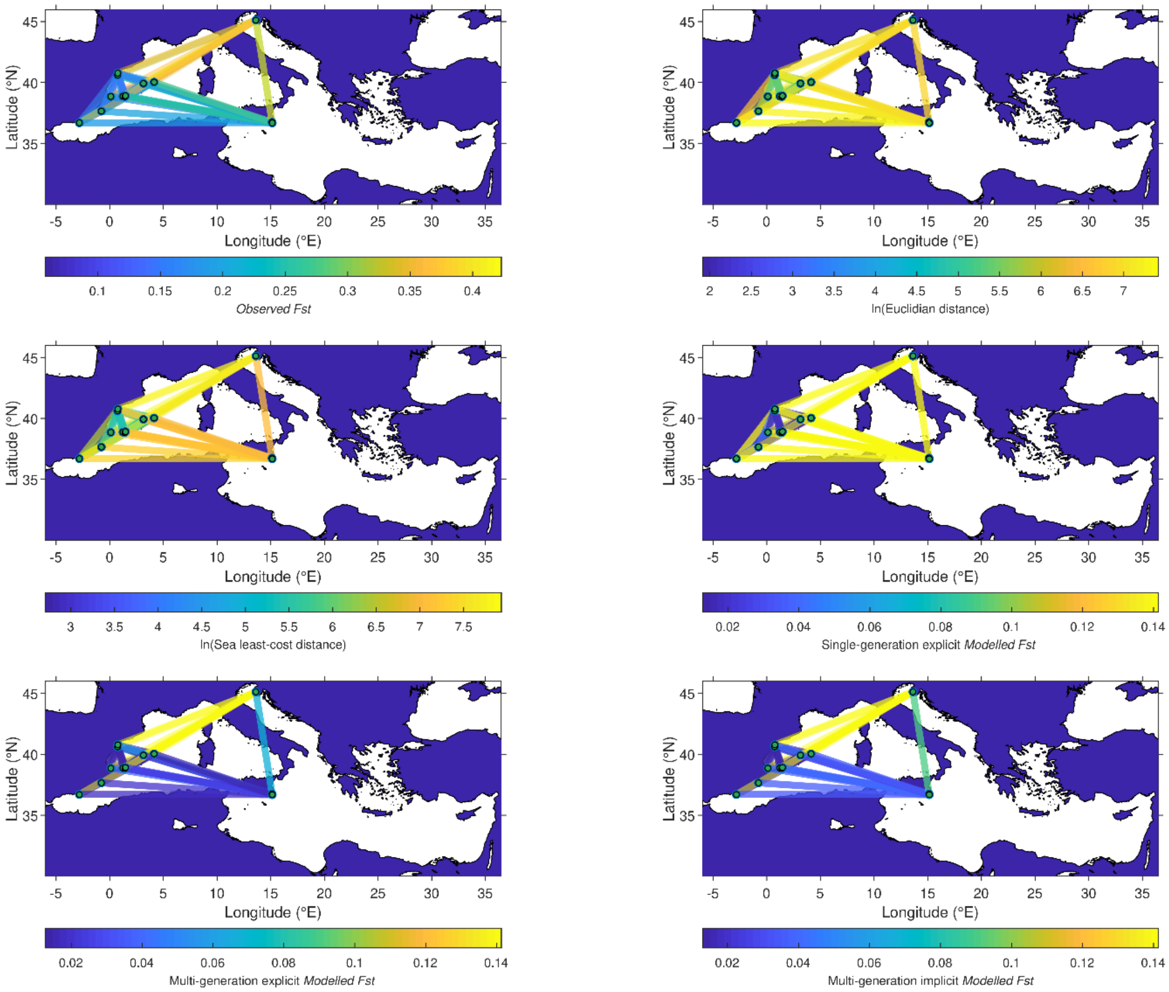


*Supplementary Figure 9:* ***Network representation of*** **observed** ***and*** **modelled** ***genetic structures*** *for* Cymodocea nodosa*; Alberto et al., (2008).* ***a*** $F_{st}^{obs}$ *values,* ***b*** *log*$_e$*(Euclidian distance),* ***c*** *log*$_e$*(Sea least-cost distance),* ***d*** *single-generation explicit* $F_{st}^{mod}$*,* ***e*** *multi-generation explicit* $F_{st}^{mod}$ *and* ***f*** *multi-generation implicit* $F_{st}^{mod}$*. Similar plots made for each study of our meta-analysis can be viewed at: https://nuage.osupytheas.fr/s/3fj5TidxC4A6Kax*

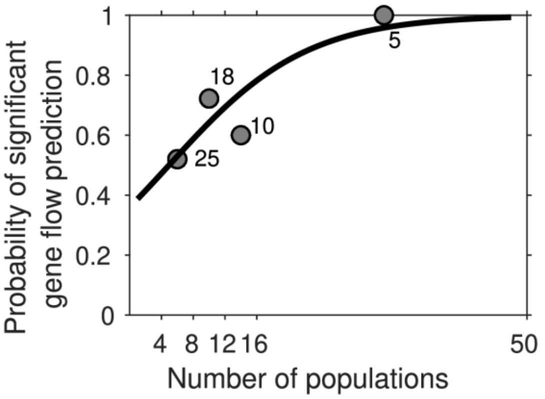


*Supplementary Figure 10:* ***Sensitivity of the multi-generation implicit dispersal model.*** *Probability of significant gene flow predictions (i.e. probability to obtain significant* $R^2$ *between* $F_{st}^{mod}$ *and* $F_{st}^{obs}/(1-F_{st}^{obs})$*) as a function of the number of sampled populations binned in four categories (4-8, 8-12,12-16 and > 16 populations sampled). The thick black line represents the logit model (*$R^2$ *= 0.72***). Source data are provided as a Source Data file.*

# Supplementary Methods 6: Statistical analyses on the impact of species and study characteristics on MLPE linear mixed model predictions

*Supplementary Table 10:* ***Sensitivity of the MLPE linear mixed model predictions of the multi-generation implicit dispersal model for the 58 population genetic studies included in the meta-analysis.*** *We test the sensitivity of the Species characteristics (taxa, PLD and spawning season) and study characteristics (Marker, $F_{st}$ range, Number of populations sampled and Spatial Sampling Representativeness) on* ***a*** *$R^2$ and* ***b*** *p-values either with ANOVA or a linear regression (after a log10 transformation of $R^2$ and p-values). Source data are provided as a Source Data file.*

a

| | MLPE linear mixed model $R^2$ | | | | |
|---|---|---|---|---|---|
| | *Characteristics* | *Statistic method* | *p-value* | *$R^2$* | *β slope* |
| Species | Taxa | ANOVA | 0.8385 | NaN | NaN |
| | PLD | ANOVA | 0.1362 | NaN | NaN |
| | Spawning season | ANOVA | 0.9624 | NaN | NaN |
| Study | Marker | ANOVA | 0.1832 | NaN | NaN |
| | $F_{st}$ range | log Regression | 0.8966 | 0.0005 | 0.0097 |
| | Nbr of populations | log Regression | 0.0988 | 0.0759 | -0.23 |
| | $D_{btw}$ | log Regression | 0.9792 | 0 | 0.0056 |

b

| | MLPE linear mixed model p-value | | | | |
|---|---|---|---|---|---|
| | *Characteristics* | *Statistic method* | *p-value* | *$R^2$* | *β slope* |
| Species | Taxa | ANOVA | 0.1453 | NaN | NaN |
| | PLD | ANOVA | 0.2092 | NaN | NaN |
| | Spawning season | ANOVA | 0.846 | NaN | NaN |
| Study | Marker | ANOVA | 0.6118 | NaN | NaN |
| | $F_{st}$ range | log Regression | 0.0009 | 0.1788 | -10.0746 |
| | Nbr of populations | log Regression | 0 | 0.403 | -31.6825 |
| | $D_{btw}$ | log Regression | 0.9755 | 0 | -0.3246 |

# Supplementary References:


Alberto, F., Massa, S., Manent, P., Diaz-Almela, E., Arnaud-Haond, S., Duarte, C.M., Serrão, E.A., 2008. Genetic differentiation and secondary contact zone in the seagrass Cymodocea nodosa across the Mediterranean–Atlantic transition region. J. Biogeogr. 35, 1279–1294. https://doi.org/10.1111/j.1365-2699.2007.01876.x

Arculeo, M., Pellerito, R., Bonhomme, F., 2010. Isolation and use of microsatellite loci in *Melicertus kerathurus* (Crustacea, Penaeidae). Aquat. Living Resour. 23, 103–107. https://doi.org/10.1051/alr/2010008

Arnaud-Haond, S., Migliaccio, M., Diaz-Almela, E., Teixeira, S., Van De Vliet, M.S., Alberto, F., Procaccini, G., Duarte, C.M., Serrão, E.A., 2007. Vicariance patterns in the Mediterranean Sea: east–west cleavage and low dispersal in the endemic seagrass *Posidonia oceanica*. J. Biogeogr. 34, 963–976. https://doi.org/10.1111/j.1365-2699.2006.01671.x

Aurelle, D., Ledoux, J.-B., Rocher, C., Borsa, P., Chenuil, A., Féral, J.-P., 2011. Phylogeography of the red coral (Corallium rubrum): inferences on the evolutionary history of a temperate gorgonian. Genetica 139, 855–869. https://doi.org/10.1007/s10709-011-9589-6

Baeta, M., Galimany, E., Ramón, M., 2016. Growth and reproductive biology of the sea star Astropecten aranciacus (Echinodermata, Asteroidea) on the continental shelf of the Catalan Sea (northwestern Mediterranean). Helgol. Mar. Res. 70, 1. https://doi.org/10.1186/s10152-016-0453-z

Bahri-Sfar, L., Kaouèche, M., Haffani, M., Ouanes, K., Ben Hassine, O.K., 2011. Genetic population structure of the common sole, *Solea solea* Linnaeus, 1758 (Pisces, Pleuronectiformes) along the southern shores of the Mediterranean Sea (Tunisian coasts). Ital. J. Zool. 78, 157–167. https://doi.org/10.1080/11250003.2010.532513

Baldacconi, R., Nonnis-Marzano, C., Gaino, E., Corriero, G., 2007. Sexual reproduction, larval development and release in Spongia oYcinalis L. (Porifera, Demospongiae) from the Apulian coast. Mar Biol 11.

Bernardi, G., Azzurro, E., Golani, D., Miller, M.R., 2016. Genomic signatures of rapid adaptive evolution in the bluespotted cornetfish, a Mediterranean Lessepsian invader. Mol. Ecol. 25, 3384–3396. https://doi.org/10.1111/mec.13682

Bierne, N., Launey, S., Naciri-Graven, Y., Bonhomme, F., 1998. Early Effect of Inbreeding as Revealed by Microsatellite Analyses on Ostrea edulis Larvae. Genetics 148, 1893–1906.

Borrero-Pérez, G.H., González-Wangüemert, M., Marcos, C., Pérez-Ruzafa, A., 2011. Phylogeography of the Atlanto-Mediterranean sea cucumber Holothuria (Holothuria) mammata: the combined effects of historical processes and current oceanographical pattern: PHYLOGEOGRAPHY OF HOLOTHURIA MAMMATA. Mol. Ecol. 20, 1964–1975. https://doi.org/10.1111/j.1365-294X.2011.05068.x

Boscari, E., Abbiati, M., Badalamenti, F., Bavestrello, G., Benedetti-Cecchi, L., Cannas, R., Cau, A., Cerrano, C., Chimienti, G., Costantini, F., Fraschetti, S., Ingrosso, G., Marino, I.A.M., Mastrototaro, F., Papetti, C., Paterno, M., Ponti, M., Zane, L., Congiu, L., 2019. A population genomics insight by 2b-RAD reveals populations' uniqueness along the Italian coastline in *Leptopsammia pruvoti* (Scleractinia, Dendrophylliidae). Divers. Distrib. 25, 1101–1117. https://doi.org/10.1111/ddi.12918

Boyden, C.R., Russell, P.J.C., 1972. The Distribution and Habitat Range of the Brackish Water Cockle (Cardium (Cerastoderma) glaucum) in the British Isles. J. Anim. Ecol. 41, 719. https://doi.org/10.2307/3205

Caceres-Martinez, J., Robledo, J., Figueras, A., 1993. Settlement of mussels Mytilus galloprovincialis on an exposed rocky shore in Ria de Vigo, NW Spain. Mar. Ecol. Prog. Ser. 93, 195–198. https://doi.org/10.3354/meps093195

Calò, A., Muñoz, I., Pérez-Ruzafa, Á., Vergara-Chen, C., García-Charton, J.A., 2016. Spatial genetic structure in the saddled sea bream (Oblada melanura [Linnaeus, 1758]) suggests multi-scaled patterns of connectivity between protected and unprotected areas in the Western Mediterranean Sea. Fish. Res. 176, 30–38. https://doi.org/10.1016/j.fishres.2015.12.001

Cánovas-Molina, A., Montefalcone, M., Bavestrello, G., Masmoudi, M.B., Haguenauer, A., Hammami, P., Chaoui, L., Kara, M.H., Aurelle, D., 2018. From depth to regional spatial genetic differentiation of Eunicella cavolini in the NW Mediterranean. C. R. Biol. 341, 421–432. https://doi.org/10.1016/j.crvi.2018.09.002

Carlton, J.T., Cohen, A.N., 2003. Episodic global dispersal in shallow water marine organisms: the case history of the European shore crabs *Carcinus maenas* and *C. aestuarii*: Episodic global invasion patterns in shore crabs. J. Biogeogr. 30, 1809–1820. https://doi.org/10.1111/j.1365-2699.2003.00962.x

Carreras, C., García-Cisneros, A., Wangensteen, O.S., Ordóñez, V., Palacín, C., Pascual, M., Turon, X., 2020. East is East and West is West: Population genomics and hierarchical analyses reveal genetic structure and adaptation footprints in the keystone species *Paracentrotus lividus* (Echinoidea). Divers. Distrib. 26, 382–398. https://doi.org/10.1111/ddi.13016

Carreras, C., Ordóñez, V., Zane, L., Kruschel, C., Nasto, I., Macpherson, E., Pascual, M., 2017. Population genomics of an endemic Mediterranean fish: differentiation by fine scale dispersal and adaptation. Sci. Rep. 7, 43417. https://doi.org/10.1038/srep43417

Casado-Amezúa, P., Goffredo, S., Templado, J., Machordom, A., 2012. Genetic assessment of population structure and connectivity in the threatened Mediterranean coral *Astroides calycularis* (Scleractinia, Dendrophylliidae) at different spatial scales: GENETIC STRUCTURE IN *ASTROIDES CALYCULARIS*. Mol. Ecol. 21, 3671–3685. https://doi.org/10.1111/j.1365-294X.2012.05655.x

Coelho, M.A.G., Lasker, H.R., 2016. Larval Dispersal and Population Connectivity in Anthozoans, in: Goffredo, S., Dubinsky, Z. (Eds.), The Cnidaria, Past, Present and Future: The World of Medusa and Her Sisters. Springer International Publishing, Cham, pp. 291–315. https://doi.org/10.1007/978-3-319-31305-4_19

Costantini, F., Carlesi, L., Abbiati, M., 2013. Quantifying Spatial Genetic Structuring in Mesophotic Populations of the Precious Coral Corallium rubrum. PLoS ONE 8, e61546. https://doi.org/10.1371/journal.pone.0061546

Courant, R., Friedrichs, K., Lewy, H., 1928. Über die partiellen Differenzengleichungen der mathematischen Physik. Math. Ann. 100, 32–74. https://doi.org/10.1007/BF01448839

Cuesta, J.A., Rodríguez, A., 2000. Zoeal stages of the intertidal crab Pachygrapsus marmoratus (Fabricius, 1787) (Brachyura, Grapsidae) reared in the laboratory. Hydrobiologia 436, 119–130. https://doi.org/10.1023/A:1026576614590

Dailianis, T., Tsigenopoulos, C.S., Dounas, C., Voultsiadou, E., 2011. Genetic diversity of the imperilled bath sponge Spongia officinalis Linnaeus, 1759 across the Mediterranean Sea: patterns of population differentiation and implications for taxonomy and conservation: GENETIC DIVERSITY OF SPONGIA OFFICINALIS. Mol. Ecol. 20, 3757–3772. https://doi.org/10.1111/j.1365-294X.2011.05222.x

Dalongeville, A., Benestan, L., Mouillot, D., Lobreaux, S., Manel, S., 2018. Combining six genome scan methods to detect candidate genes to salinity in the Mediterranean striped red mullet (Mullus surmuletus). BMC Genomics 19, 217. https://doi.org/10.1186/s12864-018-4579-z

Di Franco, A., Guidetti, P., 2011. Patterns of variability in early-life traits of fishes depend on spatial scale of analysis. Biol. Lett. 7, 454–456. https://doi.org/10.1098/rsbl.2010.1149

Di Franco, A., Qian, K., Calò, A., Di Lorenzo, M., Planes, S., Guidetti, P., 2013. Patterns of variability in early life traits of a Mediterranean coastal fish. Mar. Ecol. Prog. Ser. 476, 227–235.

Diz, A.P., Presa, P., 2008. Regional patterns of microsatellite variation in Mytilus galloprovincialis from the Iberian Peninsula. Mar. Biol. 154, 277–286. https://doi.org/10.1007/s00227-008-0921-3

Durand, J., Blel, H., Shen, K., Koutrakis, E., Guinand, B., 2013. Population genetic structure of Mugil cephalus in the Mediterranean and Black Seas: a single mitochondrial clade and many nuclear barriers. Mar. Ecol. Prog. Ser. 474, 243–261. https://doi.org/10.3354/meps10080

Ernande, B., Clobert, J., McCombie, H., Boudry, P., 2003. Genetic polymorphism and trade-offs in the early life-history strategy of the Pacific oyster, Crassostrea gigas (Thunberg, 1795): a quantitative genetic study: Early life-history strategy in a marine bivalve. J. Evol. Biol. 16, 399–414. https://doi.org/10.1046/j.1420-9101.2003.00543.x

Fassatoui, C., Mdelgi, E., Romdhane, M.S., 2009. A preliminary investigation of allozyme genetic variation and population structure in common pandora (Pagellus erythrinus, Sparidae) from Tunisian and Libyan coasts. Ichthyol. Res. 56, 301–307. https://doi.org/10.1007/s10228-008-0094-6

Félix-Hackradt, F.C., Hackradt, C.W., Pérez-Ruzafa, Á., García-Charton, J.A., 2013. Discordant patterns of genetic connectivity between two sympatric species, Mullus barbatus (Linnaeus, 1758) and Mullus surmuletus (Linnaeus, 1758), in south-western Mediterranean Sea. Mar. Environ. Res. 92, 23–34. https://doi.org/10.1016/j.marenvres.2013.08.008

Franchini, P., Sola, L., Crosetti, D., Milana, V., Rossi, A.R., 2012. Low levels of population genetic structure in the gilthead sea bream, Sparus aurata, along the coast of Italy. ICES J. Mar. Sci. 69, 41–50. https://doi.org/10.1093/icesjms/fsr175

Fratini, S., Ragionieri, L., Cutuli, G., Vannini, M., Cannicci, S., 2013. Pattern of genetic isolation in the crab Pachygrapsus marmoratus within the Tuscan Archipelago (Mediterranean Sea). Mar. Ecol. Prog. Ser. 478, 173–183. https://doi.org/10.3354/meps10247

Fruciano, C., Tigano, C., Ferrito, V., 2011. Geographical and morphological variation within and between colour phases in *Coris julis* (L. 1758), a protogynous marine fish: GEOGRAPHICAL VARIATION IN A PROTOGYNOUS FISH. Biol. J. Linn. Soc. 104, 148–162. https://doi.org/10.1111/j.1095-8312.2011.01700.x

Gaino, E., Baldacconi, R., Corriero, G., 2007. Post-larval development of the commercial sponge Spongia officinalis L. (Porifera, Demospongiae). Tissue Cell 39, 325–334. https://doi.org/10.1016/j.tice.2007.06.006

Galarza, J.A., Turner, G.F., Macpherson, E., Rico, C., 2009. Patterns of genetic differentiation between two co-occurring demersal species: the red mullet (Mullus barbatus) and the striped red mullet (Mullus surmuletus). Can. J. Fish. Aquat. Sci. 66, 1478–1490. https://doi.org/10.1139/F09-098

Garoia, F., Guarniero, I., Grifoni, D., Marzola, S., Tinti, F., 2007. Comparative analysis of AFLPs and SSRs efficiency in resolving population genetic structure of Mediterranean Solea vulgaris: EFFICIENCY OF SSRs AND AFLPs IN FISH POPULATION ANALYSIS. Mol. Ecol. 16, 1377–1387. https://doi.org/10.1111/j.1365-294X.2007.03247.x

Gharbi, A., Zitari-Chatti, R., Van Wormhoudt, A., Dhraief, M.N., Denis, F., Said, K., Chatti, N., 2011. Allozyme Variation and Population Genetic Structure in the Carpet Shell Clam Ruditapes decussatus Across the Siculo-Tunisian Strait. Biochem. Genet. 49, 788–805. https://doi.org/10.1007/s10528-011-9450-8

Gkafas, G., Tsigenopoulos, C., Magoulas, A., Panagiotaki, P., Vafidis, D., Mamuris, Z., Exadactylos, A., 2013. Population subdivision of saddled seabream Oblada melanura in the Aegean Sea revealed by genetic and morphometric analyses. Aquat. Biol. 18, 69–80. https://doi.org/10.3354/ab00490

Goffredo, S., Airi, V., Radetić, J., Zaccanti, F., 2006. Sexual reproduction of the solitary sunset cup coral Leptopsammia pruvoti (Scleractinia, Dendrophylliidae) in the Mediterranean. 2. Quantitative aspects of the annual reproductive cycle. Mar. Biol. 148, 923–931. https://doi.org/10.1007/s00227-005-0137-8

Goffredo, S., Gasparini, G., Marconi, G., Putignano, M.T., Pazzini, C., Zaccanti, F., 2010. Gonochorism and planula brooding in the Mediterranean endemic orange coral Astroides calycularis (Scleractinia: Dendrophylliidae). Morphological aspects of gametogenesis and ontogenesis. Mar. Biol. Res. 6, 421–436. https://doi.org/10.1080/17451000903428488

Goffredo, S., Mezzomonaco, L., Zaccanti, F., 2004. Genetic differentiation among populations of the Mediterranean hermaphroditic brooding coral Balanophyllia europaea (Scleractinia: Dendrophylliidae). Mar. Biol. 145, 1075–1083. https://doi.org/10.1007/s00227-004-1403-x

González-Wangüemert, M., Cánovas, F., Pérez-Ruzafa, A., Marcos, C., Alexandrino, P., 2010. Connectivity patterns inferred from the genetic structure of white seabream (Diplodus sargus L.). J. Exp. Mar. Biol. Ecol. 383, 23–31. https://doi.org/10.1016/j.jembe.2009.10.010

Hammami, I., Bahri-Sfar, L., Kaoueche, M., Hassine, O.K.B., 2007. Genetic characterization of striped sea bream (Lithognathus mormyrus) populations on both sides of a boundary area between eastern and western Mediterranean basins 5.

Hidalgo, M., Rossi, V., Monroy, P., Ser-Giacomi, E., Hernández-García, E., Guijarro, B., Massutí, E., Alemany, F., Jadaud, A., Perez, J.L., Reglero, P., 2019. Accounting for ocean connectivity and hydroclimate in fish recruitment fluctuations within transboundary metapopulations. Ecol. Appl. 0, e01913. https://doi.org/10.1002/eap.1913

Hunter, 1999. BIOLOGY OF THE EUROPEAN SPINY LOBSTER, PALINURUS ELEPHAS (FABRICIUS, 1787) (DECAPODA, PALINURIDEA). Crustaceana 72, 545–565. https://doi.org/10.1163/156854099503609

Kaouèche, M., Bahri-Sfar, L., Hammami, I., Hassine, O.K.B., 2013. Morphological and genetic variations of Diplodus vulgaris along the Tunisian coasts 10.

Kersting, D., Casado, C., López-Legentil, S., Linares, C., 2013. Unexpected patterns in the sexual reproduction of the Mediterranean scleractinian coral Cladocora caespitosa. Mar. Ecol. Prog. Ser. 486, 165–171. https://doi.org/10.3354/meps10356

Kružić, P., Žuljević, A., Nikolić, V., 2008. Spawning of the colonial coral Cladocora caespitosa (Anthozoa, Scleractinia) in the Southern Adriatic Sea. Coral Reefs 27, 337–341. https://doi.org/10.1007/s00338-007-0334-7

Kuo, C.-M., Shehadeh, Z.H., Milken, K.K., 1973. A preliminary report on the development, growth and survival of laboratory reared larvae of the grey mullet, Mugil cephalus L. J. Fish Biol. 5, 459–470. https://doi.org/10.1111/j.1095-8649.1973.tb04475.x

Launey, S., 2002. Geographic Structure in the European Flat Oyster (Ostrea edulis L.) as Revealed by Microsatellite Polymorphism. J. Hered. 93, 331–351. https://doi.org/10.1093/jhered/93.5.331

Lazoski, C., Solé-Cava, A., Boury-Esnault, N., Klautau, M., Russo, C., 2001. Cryptic speciation in a high gene flow scenario in the oviparous marine sponge Chondrosia reniformis. Mar. Biol. 139, 421–429. https://doi.org/10.1007/s002270100542

Macpherson, E., Raventos, N., 2006. Relationship between pelagic larval duration and geographic distribution of Mediterranean littoral fishes. Mar. Ecol. Prog. Ser. 327, 257–265.

Madec, G.: NEMO ocean engine, Note du Pole de modélisation, Institut Pierre-Simon Laplace (IPSL), France, No 27 ISSN No1288-1619, 2008.

Maggio, T., Lo Brutto, S., Garoia, F., Tinti, F., Arculeo, M., 2009. Microsatellite analysis of red mullet Mullus barbatus (Perciformes, Mullidae) reveals the isolation of the Adriatic Basin in the Mediterranean Sea. ICES J. Mar. Sci. 66, 1883–1891. https://doi.org/10.1093/icesjms/fsp160

Marzouk, Z., Aurelle, D., Said, K., Chenuil, A., 2017. Cryptic lineages and high population genetic structure in the exploited marine snail Hexaplex trunculus (Gastropoda: Muricidae). Biol. J. Linn. Soc. 122, 411–428. https://doi.org/10.1093/biolinnean/blx070

Masmoudi, M.B., Chaoui, L., Topçu, N.E., Hammami, P., Kara, M.H., Aurelle, D., 2016. Contrasted levels of genetic diversity in a benthic Mediterranean octocoral: Consequences of different demographic histories? Ecol. Evol. 6, 8665–8678. https://doi.org/10.1002/ece3.2490

Melià, P., Schiavina, M., Rossetto, M., Gatto, M., Fraschetti, S., Casagrandi, R., 2016. Looking for hotspots of marine metacommunity connectivity: a methodological framework. Sci. Rep. 6, 23705.

Milano, I., Babbucci, M., Cariani, A., Atanassova, M., Bekkevold, D., Carvalho, G.R., Espiñeira, M., Fiorentino, F., Garofalo, G., Geffen, A.J., Hansen, Jakob.H., Helyar, S.J., Nielsen, E.E., Ogden, R., Patarnello, T., Stagioni, M., FishPopTrace Consortium, Tinti, F., Bargelloni, L., 2014. Outlier SNP

markers reveal fine-scale genetic structuring across European hake populations ( *Merluccius merluccius* ). Mol. Ecol. 23, 118–135. https://doi.org/10.1111/mec.12568

Morales-Nin, B., Moranta, J., 2004. Recruitment and post-settlement growth of juvenile *Merluccius merluccius* on the western Mediterranean shelf. Sci. Mar. 68, 399–409. https://doi.org/10.3989/scimar.2004.68n3399

Muths, D., Rastorgueff, P.-A., Selva, M., Chevaldonné, P., 2015. Local scale connectivity in the cave-dwelling brooding fish Apogon imberbis. J. Sea Res. 95, 70–74. https://doi.org/10.1016/j.seares.2014.10.009

Oddo, P., Adani, M., Pinardi, N., Fratianni, C., Tonani, M., Pettenuzzo, D., others, 2009. A nested Atlantic-Mediterranean Sea general circulation model for operational forecasting. Ocean Sci.

Ordóñez, V., Pascual, M., Rius, M., Turon, X., 2013. Mixed but not admixed: a spatial analysis of genetic variation of an invasive ascidian on natural and artificial substrates. Mar. Biol. 160, 1645–1660. https://doi.org/10.1007/s00227-013-2217-5

Orth, R.J., Harwell, M.C., Inglis, G.J., 2006. Ecology of Seagrass Seeds and Seagrass Dispersal Processes, in: LARKUM, A.W.D., ORTH, R.J., DUARTE, C.M. (Eds.), SEAGRASSES: BIOLOGY, ECOLOGYAND CONSERVATION. Springer Netherlands, Dordrecht, pp. 111–133. https://doi.org/10.1007/978-1-4020-2983-7_5

Palero, F., Abelló, P., Macpherson, E., Beaumont, M., Pascual, M., 2011. Effect of oceanographic barriers and overfishing on the population genetic structure of the European spiny lobster ( *Palinurus elephas* ): POPULATION GENETICS OF PALINURUS. Biol. J. Linn. Soc. 104, 407–418. https://doi.org/10.1111/j.1095-8312.2011.01728.x

Pallaoro, A., Jardas, I., 2003. Some biological parameters of the peacock wrasse, *Symphodus (Crenilabrus) tinca* (L. 1758) (Pisces: Labridae) from the middle eastern Adriatic (Croatian coast). Sci. Mar. 67, 33–41. https://doi.org/10.3989/scimar.2003.67n133

Paterno, M., Schiavina, M., Aglieri, G., Souissi, J.B., Boscari, E., Casagrandi, R., Chassanite, A., Chiantore, M., Congiu, L., Guarnieri, G., Kruschel, C., Macic, V., Marino, I.A.M., Papetti, C., Patarnello, T., Zane, L., Melià, P., 2017. Population genomics meet Lagrangian simulations: Oceanographic patterns and long larval duration ensure connectivity among Paracentrotus lividus populations in the Adriatic and Ionian seas. Ecol. Evol. 7, 2463–2479. https://doi.org/10.1002/ece3.2844

Pedrotti, M.L., 1993. Spatial and temporal distribution and recruitment of echinoderm larvae in the Ligurian Sea. J. Mar. Biol. Assoc. U. K. 73, 513–530. https://doi.org/10.1017/S0025315400033075

Penant, G., Aurelle, D., Feral, J., Chenuil, A., 2013. Planktonic larvae do not ensure gene flow in the edible sea urchin Paracentrotus lividus. Mar. Ecol. Prog. Ser. 480, 155–170. https://doi.org/10.3354/meps10194

Pérez-Portela, R., Palacín, C., Duran, S., Turon, X., 2007. Biological traits of three closely related species of Pycnoclavella (Ascidiacea) in the Western Mediterranean. Mar. Biol. 152, 1031–1038. https://doi.org/10.1007/s00227-007-0750-9

Raventos, N., 2007. Age, growth and reproductive parameters of the Mediterranean cardinal fish, Apogon imberbis. J. Appl. Ichthyol. 23, 675–678. https://doi.org/10.1111/j.1439-0426.2007.00847.x

Reem, E., Douek, J., Paz, G., Katzir, G., Rinkevich, B., 2017. Phylogenetics, biogeography and population genetics of the ascidian Botryllus schlosseri in the Mediterranean Sea and beyond. Mol. Phylogenet. Evol. 107, 221–231. https://doi.org/10.1016/j.ympev.2016.10.005

Rius, M., Pineda, M.C., Turon, X., 2009. Population dynamics and life cycle of the introduced ascidian Microcosmus squamiger in the Mediterranean Sea. Biol. Invasions 11, 2181–2194. https://doi.org/10.1007/s10530-008-9375-2

Rius, M., Turon, X., Dias, G.M., Marshall, D.J., 2010. Propagule size effects across multiple life-history stages in a marine invertebrate: *Propagule size across life-history stages*. Funct. Ecol. 24, 685–693. https://doi.org/10.1111/j.1365-2435.2009.01668.x

Roberts, S.D., Dixon, C.D., Andreacchio, L., 2012. Temperature dependent larval duration and survival of the western king prawn, Penaeus (Melicertus) latisulcatus Kishinouye, from Spencer Gulf,

South Australia. J. Exp. Mar. Biol. Ecol. 411, 14–22. https://doi.org/10.1016/j.jembe.2011.10.022

Santos, R., Dias, S., Tecelão, C., Pedrosa, R., Pombo, A., n.d. Reproductive biological characteristics and fatty acid profile of Holothuria mammata (Grube, 1840) 8.

Sá-Pinto, A., Branco, M.S., Alexandrino, P.B., Fontaine, M.C., Baird, S.J.E., 2012. Barriers to Gene Flow in the Marine Environment: Insights from Two Common Intertidal Limpet Species of the Atlantic and Mediterranean. PLoS ONE 7, e50330. https://doi.org/10.1371/journal.pone.0050330

Schiavina, M., Marino, I. a. M., Zane, L., Melià, P., 2014. Matching oceanography and genetics at the basin scale. Seascape connectivity of the Mediterranean shore crab in the Adriatic Sea. Mol. Ecol. 23, 5496–5507. https://doi.org/10.1111/mec.12956

Schunter, C., Carreras-Carbonell, J., Macpherson, E., Tintoré, J., Vidal-Vijande, E., Pascual, A., Guidetti, P., Pascual, M., 2011. Matching genetics with oceanography: directional gene flow in a Mediterranean fish species. Mol. Ecol. 20, 5167–5181. https://doi.org/10.1111/j.1365-294X.2011.05355.x

Ser-Giacomi, E., Rossi, V., López, C., Hernandez-Garcia, E., 2015. Flow networks: A characterization of geophysical fluid transport. Chaos Interdiscip. J. Nonlinear Sci. 25, 036404.

Serra, I.A., Innocenti, A.M., Maida, G.D., Calvo, S., Migliaccio, M., Zambianchi, E., Pizzigalli, C., Arnaud-Haond, S., Duarte, C.M., Serrao, E.A., Procaccini, G., 2010. Genetic structure in the Mediterranean seagrass Posidonia oceanica: disentangling past vicariance events from contemporary patterns of gene flow. Mol. Ecol. 19, 557–568. https://doi.org/10.1111/j.1365-294X.2009.04462.x

Shabtay, A., Tikochinski, Y., Benayahu, Y., Rilov, G., 2014. Preliminary data on the genetic structure of a highly successful invading population of oyster suggesting its establishment dynamics in the Levant. Mar. Biol. Res. 10, 407–415. https://doi.org/10.1080/17451000.2013.814790

Soria, G., Tordecillas-Guillen, J., Cudney-Bueno, R., Shaw, W., 2010. Spawning Induction, Fecundity Estimation, and Larval Culture of *Spondylus calcifer* (Carpenter, 1857) (Bivalvia: Spondylidae). J. Shellfish Res. 29, 143–149. https://doi.org/10.2983/035.029.0108

Sromek, L., Forcioli, D., Lasota, R., Furla, P., Wolowicz, M., 2019. Next-generation phylogeography of the cockle *Cerastoderma glaucum* : Highly heterogeneous genetic differentiation in a lagoon species. Ecol. Evol. 9, 4667–4682. https://doi.org/10.1002/ece3.5070

Susini, M.-L., Thibaut, T., Meinesz, A., Forcioli, D., 2007. A preliminary study of genetic diversity in Cystoseira amentacea (C. Agardh) Bory var. stricta Montagne (Fucales, Phaeophyceae) using random amplified polymorphic DNA. Phycologia 46, 605–611. https://doi.org/10.2216/06-100.1

Teixidó, N., Garrabou, J., Harmelin, J.-G., 2011. Low Dynamics, High Longevity and Persistence of Sessile Structural Species Dwelling on Mediterranean Coralligenous Outcrops. PLoS ONE 6, e23744. https://doi.org/10.1371/journal.pone.0023744

Thibaut, T., Bottin, L., Aurelle, D., Boudouresque, C.-F., Blanfuné, A., Verlaque, M., Pairaud, I., Millet, B., 2016. Connectivity of Populations of the Seaweed Cystoseira amentacea within the Bay of Marseille (Mediterranean Sea): Genetic Structure and Hydrodynamic Connections. Cryptogam. Algol. 37, 233–255. https://doi.org/10.7872/crya/v37.iss4.2016.233

Vasconcelos, P., Gaspar, M.B., Joaquim, S., Matias, D., Castro, M., 2004. Spawning of *Hexaplex (Trunculariopsis) trunculus* (Gastropoda: Muricidae) in the laboratory: description of spawning behaviour, egg masses, embryonic development, hatchling and juvenile growth rates. Invertebr. Reprod. Dev. 46, 125–138. https://doi.org/10.1080/07924259.2004.9652616

Vaz, A.C., Scarcella, G., Pardal, M.A., Martinho, F., 2019. Water temperature gradients drive early life-history patterns of the common sole (Solea solea L.) in the Northeast Atlantic and Mediterranean. Aquat. Ecol. 53, 281–294. https://doi.org/10.1007/s10452-019-09688-2

Villamor, A., Costantini, F., Abbiati, M., 2014. Genetic Structuring across Marine Biogeographic Boundaries in Rocky Shore Invertebrates. PLOS ONE 9, e101135. https://doi.org/10.1371/journal.pone.0101135

Wanninger, A., Haszprunar, G., 2002. Chiton myogenesis: Perspectives for the development and evolution of larval and adult muscle systems in molluscs. J. Morphol. 251, 103–113. https://doi.org/10.1002/jmor.1077

Weber, A. a.-T., Mérigot, B., Valière, S., Chenuil, A., 2015. Influence of the larval phase on connectivity: strong differences in the genetic structure of brooders and broadcasters in the Ophioderma longicauda species complex. Mol. Ecol. 24, 6080–6094. https://doi.org/10.1111/mec.13456

Weber, A.A.-T., Stöhr, S., Chenuil, A., 2014. Genetic data, reproduction season and reproductive strategy data support the existence of biological species in Ophioderma longicauda. C. R. Biol. 337, 553–560. https://doi.org/10.1016/j.crvi.2014.07.007

Zitari-Chatti, R., Chatti, N., Elouaer, A., Said, K., 2007. Genetic variation and population structure of the caramote prawn Penaeus kerathurus (Forskäl) from the eastern and western Mediterranean coasts in Tunisia: Genetic variation in Penaeus kerathurus. Aquac. Res. 39, 70–76. https://doi.org/10.1111/j.1365-2109.2007.01874.x

Zulliger, D.E., Tanner, S., Ruch, M., Ribi, G., 2009. Genetic structure of the high dispersal Atlanto-Mediterreanean sea star Astropecten aranciacus revealed by mitochondrial DNA sequences and microsatellite loci. Mar. Biol. 156, 597–610. https://doi.org/10.1007/s00227-008-1111-z
