# Supplementary material for: Spatial coalescent connectivity through multi-generation dispersal modelling predicts gene flow across marine phyla
Source: Nat Commun. 2022 Oct 4;13:5861. doi: 10.1038/s41467-022-33499-z (PMC9532449; doi:10.1038/s41467-022-33499-z)
Supplement: Supplementary file 3 — Description of Additional Supplementary Files [file 41467_2022_33499_MOESM3_ESM.pdf]

Description of Additional Supplementary Files

File Name: Supplementary Dataset 1

Description: Genetic differentiation between population pairs for 58 genetic population studies, encompassing 47 marine species classified in nine taxonomic groups. 'ID' refers to the study ID (i.e. from 1 to 58). 'First_author' refers to the name of the first author of the study (complete reference can be found in Supplementary Table 1). 'Year' refers to the year of publication of the first author of the study (complete reference can be found in Supplementary Table 1). 'Taxa' refers to the taxonomic group (Algae, Anthozoa, Ascidiacea, Crustacea, Demospongiae, Echinodermata, Fish, Mollusca and Phanerogam). 'Species' refers to the model species of each published study. 'Long1' refers to the longitude of the sampled population A. 'Lat1' refers to the latitude of the sampled population A. 'Long2' refers to the longitude of the sampled population B. 'Lat2' refers to the latitude of the sampled population B. 'Marker' refers to the genetic marker used (allozymes, microsatellites, mitochondrial DNA sequences, nuclear DNA sequences and SNPs from high throughput sequencing). 'Fst' refers to the measure of population differentiation between population A and population B. 'p_value' refers to the p-value associated with the Fst calculation.
