# Supplementary material for: Spatial coalescent connectivity through multi-generation dispersal modelling predicts gene flow across marine phyla
Source: Nat Commun. 2022 Oct 4;13:5861. doi: 10.1038/s41467-022-33499-z (PMC9532449; doi:10.1038/s41467-022-33499-z)
Supplement: Supplementary file 5 — Reporting Summary [file 41467_2022_33499_MOESM5_ESM.pdf]

nature portfolio

Corresponding author(s): Térence Legrand; Vincent Rossi

Last updated by author(s): Aug 19, 2022

Reporting Summary

Nature Portfolio wishes to improve the reproducibility of the work that we publish. This form provides structure for consistency and transparency in reporting. For further information on Nature Portfolio policies, see our Editorial Policies and the Editorial Policy Checklist.

Statistics

For all statistical analyses, confirm that the following items are present in the figure legend, table legend, main text, or Methods section.

n/a	Confirmed	
☐	☒	The exact sample size (*n*) for each experimental group/condition, given as a discrete number and unit of measurement
☐	☒	A statement on whether measurements were taken from distinct samples or whether the same sample was measured repeatedly
☐	☒	The statistical test(s) used AND whether they are one- or two-sided<br>*Only common tests should be described solely by name; describe more complex techniques in the Methods section.*
☒	☐	A description of all covariates tested
☒	☐	A description of any assumptions or corrections, such as tests of normality and adjustment for multiple comparisons
☐	☒	A full description of the statistical parameters including central tendency (e.g. means) or other basic estimates (e.g. regression coefficient) AND variation (e.g. standard deviation) or associated estimates of uncertainty (e.g. confidence intervals)
☐	☒	For null hypothesis testing, the test statistic (e.g. *F*, *t*, *r*) with confidence intervals, effect sizes, degrees of freedom and *P* value noted<br>*Give P values as exact values whenever suitable.*
☒	☐	For Bayesian analysis, information on the choice of priors and Markov chain Monte Carlo settings
☒	☐	For hierarchical and complex designs, identification of the appropriate level for tests and full reporting of outcomes
☐	☒	Estimates of effect sizes (e.g. Cohen's *d*, Pearson's *r*), indicating how they were calculated

Our web collection on statistics for biologists contains articles on many of the points above.

Software and code

Policy information about availability of computer code

Data collection	Data are collected with Microsoft Excel (version 2206)
Data analysis	The Lagrangian Flow Network model is coded in C++.<br>Multi-generation explicit and implicit dispersal probabilities are computed with Python (version 3.7.3). The codes have been already published (Ser-Giacomi et al. 2021 ; doi:10.1103/PhysRevE.103.042309) and are available online here: https://github.com/serjaaa/cumulated-net-conn<br>Sea least-cost distances are calculated thanks to the Marmap package (version 1.0.4) with R (version 4.0.2).<br>MLPE linear mixed models are computed thanks to the 'lmer' function of the lmerTest package (version 3.1.3) with R (version 4.0.2).<br>The coefficient of determination R^2 is computed thanks to the function 'r.squaredGLMM' of the MuMIn package (version 1.43.17) with R (version 4.0.2).<br>Mantel tests are computed thanks to the function 'mantel' of the vegan package (version 2.5-7) with R (version 4.0.2).<br>Sensitivity tests are performed using the Matlab software (version 9.4).<br>Mantel correlations comparaison are performed using the package COCOR (version 1.1-3) with R (version 4.0.5).

For manuscripts utilizing custom algorithms or software that are central to the research but not yet described in published literature, software must be made available to editors and reviewers. We strongly encourage code deposition in a community repository (e.g. GitHub). See the Nature Portfolio guidelines for submitting code & software for further information.

Data

Policy information about availability of data

All manuscripts must include a data availability statement. This statement should provide the following information, where applicable:

- Accession codes, unique identifiers, or web links for publicly available datasets
- A description of any restrictions on data availability
- For clinical datasets or third party data, please ensure that the statement adheres to our policy

The population genetic data generated in this study is provided as a Supplementary Information (genetic_differentiation_dataset.csv). Genetic population studies references are provided as Supplementary Information, as well as literature references used to configure species characteristics. We also use FishBase (Froese and Pauly 2000 ; https://www.fishbase.se/search.php) and Doris (Willis et al., 2016 ; https://doris.ffessm.fr/) webpages for global information about the species of interest. Source and raw data relevant for each figure are provided with this paper.

Human research participants

Policy information about studies involving human research participants and Sex and Gender in Research.

Reporting on sex and gender	This study does not involve human research participants
Population characteristics	This study does not involve human research participants
Recruitment	This study does not involve human research participants
Ethics oversight	This study does not involve human research participants

Note that full information on the approval of the study protocol must also be provided in the manuscript.

Field-specific reporting

Please select the one below that is the best fit for your research. If you are not sure, read the appropriate sections before making your selection.

☐ Life sciences ☐ Behavioural & social sciences ☒ Ecological, evolutionary & environmental sciences

For a reference copy of the document with all sections, see nature.com/documents/nr-reporting-summary-flat.pdf

Ecological, evolutionary & environmental sciences study design

All studies must disclose on these points even when the disclosure is negative.

Study description	The study consists in comparing genetic differentation observations (i.e. quantitative data obtained thanks to a compilation of 58 published population genetic studies) against gene flow predictions (i.e. quantitative data, obtained with five different models). They are: Isolation-By-Distance model considering Euclidian or sea-least cost distances, single-generation dispersal model simulating filial connectivity, multi-generation dispersal model simulating filial connectivity and multi-generation dispersal model simulating coalescent connectivity. Single-generation and multi-generation dispersal models are based on connectivity matrices generated by a state-of-the-art Lagrangian model whose characteristics are fully described in the Methods section and have been published elsewhere. All modelling approaches have been parameterized following the main ecological traits of all marine taxa comprised in the meta-analysis. General mathematical expressions that allow computing filial and coalescent connections are referenced in the Methods section and have been published elsewhere.
Research sample	The research sample consists in 559 sampled populations of 47 phylogenetically divergent marine sessile species distributed in nine taxonomic groups (Algae, Anthozoa, Ascidiacea, Crustacea, Demospongiae, Echinodermata, Fish, Mollusca and Phanerogam) spanning the entire Mediterranean basin. More specifically, our compilation originates from 58 published studies (the PRISMA flow chart in the Supplementary Information summarizes our compilation rational), corresponding to 3821 estimates of genetic differentiation (e.g. Fst) between population pairs.
Sampling strategy	For the meta-analysis, the authors selected among all population genetic studies those matching the following criteria: (i) its target species is poorly mobile, i.e. that is characterized by a biphasic life cycle with early-life free-swimming dispersing propagules (e.g. seeds, eggs, larvae or body fragments) and sessile to sedentary adult behaviour, and (ii) it must present at least four distinct sampled populations with at least 15 replicate individuals sampled in each of them. A threshold of n = 15 individuals per population was imposed as a trade-off to retain a large enough number of studies, while minimizing the probability of type II errors associated to limited sample size and consequently low statistical power. A threshold of n = 4 sampled populations per study (i.e. leading to 6 pairwise comparisons per studies) was imposed as a trade-off to retain a large enough number of studies, while maintaining sufficient statistical power to compare genetic differentiation observations against gene flow predictions. In a few cases, when populations were close, that is aggregated in a same bio-physical modelling locality (i.e. ¼° sub-areas) we eliminated the study.
Data collection	The authors (A.C, S.A-H. and T.L.) collected the data from 2002 to 2015 by selecting studies which fulfilled the two criteria described

	above in a previous review of genetic differentiation (Porro et al., 2015). Authors (A.C. and T.L.) collected the data from 2015 to 2020 via database (Web of Sciences) and other methods (specific knowledge of the literature) by selecting studies which fulfilled the two criteria described above and are characterized by a rich dataset (i.e. population genomics). Data have been recorded on a Microsoft excel (version 2206) spreadsheet.
Timing and spatial scale	The authors (A.C, S.A-H. and T.L.) collected the data from 2002 to 2015 by selecting studies which fulfilled the two criteria described above in a previous review of genetic differentiation in the Mediterranean Sea (Porro et al., 2019). Authors (A.C. and T.L.) collected the data from 2015 to 2020 (i.e. commencement of bio-physical modelling implementation) via database (Web of Sciences) and other methods (specific knowledge of the literature) by selecting studies which fulfilled the two criteria described above and are characterized by a rich dataset (i.e. population genomics). About the spatial scale, populations must be sampled in the Mediterranean Sea and not all aggregated in a same bio-physical modelling locality (i.e. ¼° sub-areas).
Data exclusions	The authors excluded in the meta-analysis compilation all population genetic studies that do not fullfill the above-mentioned criteria (see "Sampling strategy" and the PRISMA flowchart in the Supplementary Information).
Reproducibility	No field nor lab experiments were conducted in this study, which relies on already-published data. The dataset consists in a compilation of published population genetics studies (provided in Supplementary Information) and the study uses deterministic models, ensuring reproducibility.
Randomization	Based on an exhaustive literature review (fully described in Supplementary Information)), the 47 species were allocated into coherent groups according to their main dispersal traits and favorable habitats. Due to our trait-based categorization, randomization is not relevant.
Blinding	Blinding was not relevant to our study since it does not rely on field work, lab experiments, or any other data collection procedures involving influentiable participants.

Did the study involve field work? ☐ Yes ☒ No

Reporting for specific materials, systems and methods

We require information from authors about some types of materials, experimental systems and methods used in many studies. Here, indicate whether each material, system or method listed is relevant to your study. If you are not sure if a list item applies to your research, read the appropriate section before selecting a response.

Materials & experimental systems

n/a	Involved in the study
☒	☐ Antibodies
☒	☐ Eukaryotic cell lines
☒	☐ Palaeontology and archaeology
☒	☐ Animals and other organisms
☒	☐ Clinical data
☒	☐ Dual use research of concern

Methods

n/a	Involved in the study
☒	☐ ChIP-seq
☒	☐ Flow cytometry
☒	☐ MRI-based neuroimaging
